# CRP-Mediated Carbon Catabolite Regulation of *Yersinia pestis* Biofilm Formation Is Enhanced by the Carbon Storage Regulator Protein, CsrA

**DOI:** 10.1371/journal.pone.0135481

**Published:** 2015-08-25

**Authors:** Stephan P. Willias, Sadhana Chauhan, Chien-Chi Lo, Patrick S. G. Chain, Vladimir L. Motin

**Affiliations:** 1 Department of Pathology, University of Texas Medical Branch, Galveston, Texas, United States of America; 2 Department of Microbiology & Immunology, University of Texas Medical Branch, Galveston, Texas, United States of America; 3 Bioscience Division, Los Alamos National Laboratory, Los Alamos, New Mexico, United States of America; East Carolina University School of Medicine, UNITED STATES

## Abstract

The natural transmission of *Yersinia pestis* is reliant upon biofilm blockage of the flea vector. However, the environmentally-responsive adaptive regulators which facilitate *Y*. *pestis* biofilm production in accordance with the flea midgut milieu are not well understood. We seek to establish the impact of available carbon source metabolism and storage upon *Y*. *pestis* biofilm production. Our findings demonstrate that *Y*. *pestis* biofilm production is subject to carbon catabolite regulation in which the presence of glucose impairs biofilm production; whereas, the sole metabolism of alternate carbon sources promotes robust biofilm formation. This observation is facilitated by the cAMP receptor protein, CRP. In accordance with a stark growth defect, deletion of *crp* in both CO92 and KIM6+ *Y*. *pestis* strains significantly impaired biofilm production when solely utilizing alternate carbon sources. Media supplementation with cAMP, a small-molecule activator of CRP, did not significantly alter *Y*. *pestis* biofilm production. Furthermore, CRP did not alter mRNA abundance of previously-characterized *hms* biofilm synthesis and regulation factors. Therefore, our findings indicate CRP does not confer a direct stimulatory effect, but may indirectly promote *Y*. *pestis* biofilm production by facilitating the alternate carbon source expression profile. Additionally, we assessed the impact of the carbon storage regulator protein, CsrA, upon *Y*. *pestis* biofilm production. Contrary to what has been described for *E*. *coli*, *Y*. *pestis* biofilm formation was found to be enhanced by CsrA. Regardless of media composition and available carbon source, deletion of *csrA* significantly impaired *Y*. *pestis* biofilm production. CsrA was found to promote *Y*. *pestis* biofilm production independent of glycogen regulation. Loss of *csrA* did not significantly alter relative *hmsH*, *hmsP*, or *hmsT* mRNA abundance. However, deletion of *hmsP* in the *csrA*-deficient mutant enabled excessive biofilm production, suggesting CsrA enables potent *Y*. *pestis* biofilm production through cyclic diguanylate regulation.

## Introduction

*Yersinia pestis*, the etiological agent of plague, is a historically profound pathogen responsible for three pandemics [1]. With a global distribution of endemic foci, plague remains an issue today and is considered to be a re-emerging infectious disease [2]. *Y*. *pestis* naturally circulates amongst rodent reservoir hosts and flea vectors. There are two accepted modes of *Y*. *pestis* flea-borne transmission: 1) early-phase transmission and 2) biofilm-mediated proventricular blockage. Early-phase transmission of plague, through an undetermined mechanical and/or biological process, enables rapid biofilm-independent dissemination which is suggested to contribute toward epizootic spread [3, 4]. Alternately, the zoonotic maintenance of plague may be facilitated through biofilm-mediated proventricular blockage [5, 6, 7]. Upon consumption of an infected mammalian blood meal by a naïve flea, *Y*. *pestis* forms a biofilm in association with the flea proventriculus. The biofilm prevents the passage of blood during subsequent feeding attempts and enhances regurgitation of *Y*. *pestis* into the mammalian dermis, thereby promoting the transmission of plague [8].

The *Y*. *pestis* biofilm is comprised of poly-β-1,6-*N*-acetyl-D-glucosamine (PNAG) synthesized by protein products of the *hmsHFRS* operon [9, 10]. The catalytic activity of the HmsHFRS system is subject to post-translational regulation in response to the bis-(3′,5′)-cyclic dimeric GMP (cyclic diguanylate/c-di-GMP) second messenger [11]. Cyclic diguanylate, synthesized by HmsT and HmsD diguanylate cyclases, stimulates HmsR activity [12, 13]. Conversely, c-di-GMP is degraded by the HmsP phosphodiesterase [14, 15]. However, the adaptive mechanisms which enable *Y*. *pestis* biofilm formation in response to the flea midgut milieu are not well understood.

During infection of the flea vector, components of the *Y*. *pestis* phosphoenolpyruvate (PEP): carbohydrate phosphotransferase system (PTS) (primary) carbon source metabolic pathways are notably down-regulated; whereas, the expression of non-PTS (alternate) carbon source metabolic pathways are induced [16]. The cAMP receptor protein (CRP), a central mediator of carbon catabolite repression, facilitates the metabolism of alternate carbon sources when available glucose is scarce [17]. Moreover, CRP is a global regulator which differentially modulates more than 6% of *Y*. *pestis* total protein-encoding capacity [18]. CRP functions as a DNA-binding transcription factor capable of directly promoting gene transcription. Alternately, CRP indirectly regulates gene expression via differential modulation of small, non-coding RNA (sRNA) species.

CRP has been characterized to control the expression of two competitive carbon storage system sRNA regulators, CsrB and CsrC. Studies with *Y*. *pseudotuberculosis crp*-deficient mutants demonstrate that CRP represses CsrB whilst enhancing CsrC expression [19]. Both CsrB and CsrC sRNA species bind and sequester the RNA-associating carbon storage regulator protein, CsrA [20, 21]. Through association with mRNA 5’ UTR GGA motifs, CsrA regulates gene expression by controlling transcript stability and/or translation of target transcripts [22]. CsrA has been well-documented to regulate *E*. *coli* biofilm formation through a variety of modes.

Foremost, CsrA represses *E*. *coli* glycogen synthesis which has been suggested to confer dual roles upon biofilm regulation [23, 24, 25]. Glycogen may serve as a stationary phase energy source, thereby enhancing biofilm production [26]. Alternately, through competition for common metabolic intermediates, glycogen synthesis may repress biofilm formation [27]. CsrA has also been shown to impair biofilm production by inhibiting translation of the *Y*. *pestis hmsHFRS* homolog, *pgaABCD* [28]. Moreover, CsrA disrupts mRNA transcript stability of the *E*. *coli* diguanylate cyclase *ycdT* as well as the c-di-GMP-specific phosphodiesterase *yliE* [29]. Finally, CsrA has been shown to enhance transcript stability and activate *flhDC* expression, encoding a master regulator of flagellum biosynthesis, thereby promoting motility [30]. In *Y*. *pestis*, *csrA* has been shown to be highly expressed during infection of the flea vector [16]. However, in accordance with the objective of this study, the impact of carbon metabolism and storage upon *Y*. *pestis* biofilm formation has not been ascertained.

Our findings reveal that *Y*. *pestis* biofilm formation is differentially modulated by available carbon sources: glucose inhibits biofilm formation; whereas, the metabolism of alternate carbon sources enables robust biofilm production. Scarless deletion of *crp* in both KIM6+ and CO92 *Y*. *pestis* strains significantly reduced biofilm formation relative to the respective isogenic controls. Media supplementation with cAMP, an allosteric activator of CRP, had no significant impact upon biofilm formation of either the *crp*-deficient mutants or the parental strains. Relative mRNA abundance of previously characterized Hms system biofilm-related genes were not affected by *crp*. Thus, we conclude that CRP indirectly enhances *Y*. *pestis* biofilm production by facilitating the alternate carbon source metabolism expression profile.

Contrary to what has been described for *E*. *coli*, our findings demonstrate that *Y*. *pestis* biofilm formation is enhanced by CsrA. Deletion of *csrA* in both KIM6+ and CO92 *Y*. *pestis* strains significantly impaired *Y*. *pestis* biofilm production and Congo red assimilation. The mechanism by which CsrA promotes *Y*. *pestis* biofilm production was found to be independent of glycogen regulation. Rather, deletion of *hmsP*, encoding a cyclic diguanylate phosphodiesterase, restored excessive biofilm production of the *Y*. *pestis csrA*-deficient mutant. Deletion of *csrA* did not alter *hmsP* or *hmsT* mRNA abundance relative to the respective parental strains. Nonetheless, CsrA may promote *Y*. *pestis* biofilm production through post-transcriptional maintenance of the cyclic diguanylate small molecule activator of *Y*. *pestis* biofilm synthesis.

## Materials and Methods

### Bacterial Strains, Plasmids, and Primers

Bacterial strains and plasmids used in this study are described in Table 1. Oligonucleotides are detailed in S1 Table.

**Table 1:** Bacterial Strains and Plasmids.

***Y*. *pestis* strains**	Characteristics*	**Reference**
CO92	Pgm+ pCD+	[48]
CO92 pgm-	Pgm- pCD+	Motin collection
CO92Δ*crp*	Scarless deletion of the *crp* gene	This study
CO92Δ*csrA* 5a	Scarless deletion of the *csrA* gene clone 5a	This study
CO92Δ*csrA* 3b	Scarless deletion of the *csrA* gene clone 3b	This study
CO92Δ*csrA csrA*’ 5a	Scarless Restoration of the *csrA* gene of clone 5a	This study
CO92Δ*csrA csrA*’ 3b	Scarless Restoration of the *csrA* gene of clone 3b	This study
CO92Δ*glgCAP*	Scarless deletion of the *glgCAP* gene	This study
CO92Δ*csrA* Δ*glgCAP*	Scarless deletion of the *glgCAP* gene from CO92Δ*csrA* 5a	This study
CO92Δ*hmsP*	Scarless deletion of the *hmsP* gene	This study
CO92Δ*csrA* Δ*hmsP*	Scarless deletion of the *hmsP* gene from CO92Δ*csrA* 5a	This study
KIM6+	Pgm+ pCD^−^	Brubaker collection
KIM6+ pgm-	Pgm- pCD^−^	Motin collection
KIM6+Δ*crp*	Scarless deletion of the *crp* gene	This study
KIM6+Δ*csrA* 2:14	Scarless deletion of the *csrA* gene clone 2:14	This study
KIM6+Δ*csrA* 4:12	Scarless deletion of the *csrA* gene clone 4:12	This study
KIM6+Δ*csrA csrA*’ 2:6	Scarless restoration of the *csrA* gene in clone 2:14	This study
***E*. *coli* strains**	**Characteristics**	**Reference**
DH5α	F- φ80lacZΔM15 Δ(lacZYA-argF) U169 recA1 endA1 hsdR17 (rk-, mk+) gal- phoA supE44 λ- thi-1 gyrA96 relA1	Invitrogen (Carlsbad, CA)
**Plasmids**	**Characteristics**	**Reference**
pKD46	Mutagenesis helper plasmid, source of Lambda Red Recombinase, Ap^R^	[31]
pKD4_Km-sacB	Cloned *sacB* from pCVD442 in NgoMIV site of pKD4, source of *kan-sacB* cassette, Km^R^, Ap^R^, Suc^S^	[33]
pBluescript SK+	Cloning vector, Ap^R^	Stratagene (La Jolla, CA)
pBluescript/*crp*	Cloned *crp* gene derived from KIM6+ into pBluescript SK+, Ap^R^	This study

* All *Y*. *pestis* strains contained the pMT1 and pPCP plasmids.

### Scarless Gene Deletion

The *crp* and *csrA* genes were excised from *Y*. *pestis* KIM6+ and CO92 backgrounds in a scarless fashion (S1 Fig) [31, 32, 33]. To do so, the counter-selective *sacB* gene was incorporated alongside the kanamycin resistance *kan* gene in pKD4 to obtain pKD4_Km-sacB plasmid [33]. The target gene was replaced with the combined kanamycin-resistance and sucrose sensitivity (Km^R^/Suc^S^) deletion construct via Lambda Red Recombinase mediated crossover recombination in electrocompetent *Y*. *pestis* expressing the pKD46 helper plasmid. A secondary recombination event was employed to expel the deletion construct with a customized commercial gBlock (IDT, San Jose, CA) comprised of the upstream/downstream sequences directly flanking the gene of interest, thereby scarlessly excising the gene of interest. Additional recombination events were utilized to chromosomally restore the *csrA* gene via electroporation of a PCR fragment derived by the gBlock amplification primers using CO92 DNA as a template. Verification of gene deletion was afforded by sequencing. For all mutants, retention of the *pgm* locus was confirmed via PCR of the *hmsHFRS* operon. Plasmid complementation of *crp* was afforded by incorporation into the pBluescript II SK(+) expression vector.

### Growth Kinetics

Bacteria from glycerol stocks were cultured on Heart Infusion Agar (HIA) plates for 18–24 hours at 26°C. Bacteria were sub-cultured in 25 ml Hearth Infusion Broth (HIB) at 26°C for 12–18 hours shaking at 250 rpm. 70 ml of fresh HIB was inoculated with OD_600_ = 0.2 and grown for 3 hours at 26°C whilst shaking at 250 rpm to obtain actively growing bacteria. Cultures were centrifuged at 1.8K x g for 15 min at 26°C. Residual media was aspirated and the bacteria were washed in potassium (K)-phosphate buffer (pH = 7.2). Bacteria were re-suspended to an OD_600_ = 1.0 in K-phosphate buffer. Twenty-five milliliters of either HIB or chemically defined Best Case Scenario (BCS) media supplemented with 0.2% of designated carbon sources were inoculated to an OD_600_ = 0.1 and incubated at 26°C whilst shaking at 250 rpm [34]. One milliliter aliquots were extracted at 0, 2, 6, 12, 24, 48, and 72 hours post-inoculation for optical density determination. Statistical significance was determined by repeated measures ANOVA post-hoc analysis (Tukey’s HSD P < 0.05).

### Crystal Violet Biofilm Quantification Assay

Biofilm formation was quantified via crystal violet assay [33]. Bacterial suspensions of OD_600_ = 1.0 in K-phosphate buffer were performed as previously described for the growth kinetic experiments. 24-well polystyrene plates were inoculated with 4–6 replicates of 0.75 ml of HIB or BCS supplemented with 0.2% of designated carbon sources containing OD_600_ = 0.1 bacteria. Plates were incubated at 26°C for 24 or 72 hours whilst shaking at 250 rpm. Media was aspirated, wells were washed with dH_2_O, and bacteria retained in the biofilms were stained with 0.01% crystal violet for 20 min. Crystal violet dye was aspirated and wells were washed three times with dH_2_O. Bound crystal violet was solubilized with 1.5 ml of 33% acetic acid while shaking at 150 rpm for 15 min. Absorbance was measured utilizing a BioTek Synergy HT (BioTek, Winooski, VT) at 570 nm. In order to augment CRP activity, either BCS medium utilizing 0.2% glucose as the sole carbon sources or HIB medium were supplemented with 3 mM cAMP. In all cases, error bars reflect standard deviation from the mean of two independent experiments, each consisting of 4–6 technical replicates. Statistical significance was determined by one-way ANOVA (Tukey’s HSD P <0.05).

Due to the excessive biofilm production of the *hmsP*-deficient mutants, accurate optical density readings could not be obtained during growth at 26°C. Since *Y*. *pestis* does not synthesize biofilms at temperatures >34°C, experimental pre-growth conditions were increased to 37°C in order to obtain accurate inoculums [35].

### Congo Red Binding Assay

Bacteria derived from glycerol stocks were cultured on HIA plates for 24–48 hours at 26°C. The bacteria were transferred to fresh HIA and incubated at 26°C for an additional 18–24 hours. Thereafter, 50 μL of fresh HIB was suspended to OD_600_ = 0.3. Four microliters of this suspension was spotted on Congo red plates containing either 0.2% galactose or 0.2% potassium (K)-gluconate [36]. After 48 hours or 72 hours of incubation at 26°C, Congo red assimilation was phenotypically determined.

### Semi-Quantitative Reverse Transcriptase PCR (RT-PCR)

The Access RT-PCR System (Promega, Madison, WI) was utilized to compare the mRNA abundance of previously characterized biofilm-related genes amongst CO92 and CO92Δ*crp* during growth in HIB medium as well as between CO92 cultured in chemically defined media supplemented with either 0.2% glucose or 0.2% K-gluconate. Cultures were pre-grown as previously described. 300 ml of the appropriate media was inoculated with OD_600_ = 0.075 of bacteria in 1 L flask. The culture was incubated at 26°C while shaking at 250 rpm for 6 hours. At this time, 4.0x10^9^ cells per sample as determined by optical density were added to an equal volume of RNA later (Ambion, Austin, TX). RNA was isolated via the RNAeasy midi column kit (Qiagen, Hilden, Germany) in accordance with manufacturer guidelines and processed by the TURBO DNA-free kit (Ambion) for DNase treatment. RNA concentration was determined through application of the Synergy HT Take3 Multi-Volume Plate (BioTek). RT-PCR reactions were performed in accordance with manufacturer guidelines using 25 ng of total RNA for 25 PCR cycles. Three microliter aliquots of the RT-PCR reactions were visualized on 2% agarose gels infused with ethidium bromide. Semi-quantitative analyses were performed by ImageJ software with normalization to the *gyrB* gene [33, 37, 38].

### Quantitative Real-Time PCR (qPCR)

The iQ SYBR Green Supermix (Bio-Rad, Hercules, CA) was utilized to assess relative transcript levels of *hmsH*, *hmsP*, and *hmsT* among the *csrA*-deficient mutants and the respective isogenic controls. Cultures were pre-grown in BCS medium supplemented with 0.2% K-gluconate for 18 hours incubating at 26°C while shaking at 250 rpm. Bacteria were inoculated in fresh BCS medium containing 0.2% K-gluconate and grown to logarithmic phase. Fifty milliliters of BCS medium supplemented with 0.2% K-gluconate was inoculated to an OD_600_ = 0.2. Following 6 hours of incubation at 26°C while shaking at 250 rpm, approximately 5 x 10^8^ cells per sample as determined by optical density were added to an equal volume of RNA later (Ambion). RNA was isolated via the RNAeasy midi column kit (Qiagen) in accordance with manufacturer guidelines and processed by the TURBO DNA-free kit (Ambion) for DNase treatment. For each sample, two cDNA samples were prepared by iScript cDNA Synthesis Kit (Bio-Rad) from 200 ng of total RNA as determined by Synergy HT Take3 Multi-Volume Plate (BioTek). Real-time qPCR was performed with the CFX96 Real-Time System in conjunction with the C1000 Thermal Cycler (Bio-Rad). Relative mRNA abundance was calculated by the ΔΔC_T_ Method with normalization to the *gyrB* reference gene [39]. Statistical significance was determined by one-way ANOVA comparing the average of the 2 independent cDNA samples per strain, each consisting of 3 technical replicates per optimal input concentration as dictated by prior validation.

### Whole Genome Sequencing

Whole-genome sequences of the KIM6+ and CO92 *csrA*-deficient mutants were determined (accession numbers SRS872074 and SRS872077, respectively). Bacteria derived from glycerol stocks were cultured on HIA plates for 24–48 hours at 26°C. The bacteria were transferred to fresh HIA and incubated at 26°C for an additional 18–24 hours. Genomic DNA was isolated via QIAmp DNA Mini kit (Qiagen) in accordance with manufacturer guidelines. Illumina MiSeq sequencing performed at Los Alamos National Laboratory yielded 7156350 and 8122124 respective 251 bp reads for the aforementioned strains. The Illumina reads were de novo assembled by IDBA_UD (v1.1.1) with parameter “—mink 27—maxk 127—step 20—pre_correction”. The assembled contigs were aligned to corresponding reference genomes (*Y*.*pestis* CO92, NC_003143 and *Y*.*pestis* KIM 10, NC_004088) by MUMmer (v3.23) with parameters “—maxmatch” and the uncovered reference regions were extract out using custom Perl script. Moreover, Illumina paired reads were aligned to the corresponding reference genome (*Y*.*pestis* CO92 and *Y*.*pestis* KIM 10) by BWA (v0.7.10) aln algorithm with default parameters. The average depth of coverage and percent contig recovery were calculated from alignment results and the uncovered reference regions were extract out using custom Perl script. Population variation was judged from mapping result using SAMtools (v1.1) for conversion of BWA output format to BAM format and to perform SNP and Indel analysis. The SNP calls were filtered further by vcffilter.pl of SAMtools with following criteria: (i) minimum RMS mapping quality for SNPs [10]; (ii) minimum read depth [10]; (iii) maximum read depth [10000]; (iv) minimum number of alternate bases [3]; (v) SNP within INT bp around a gap to be filtered [3]; (vi) window size for filtering adjacent gaps [10]; (vii) min P-value for strand bias (given PV4) [0.0001]; (viii) min P-value for end distance bias [0.0001]; (x) variants in the repeats region of the reference are filtered.

## Results

### Growth Kinetics of the *Y*. *pestis crp* and *csrA* Deletion Mutants

**Fig 1:**
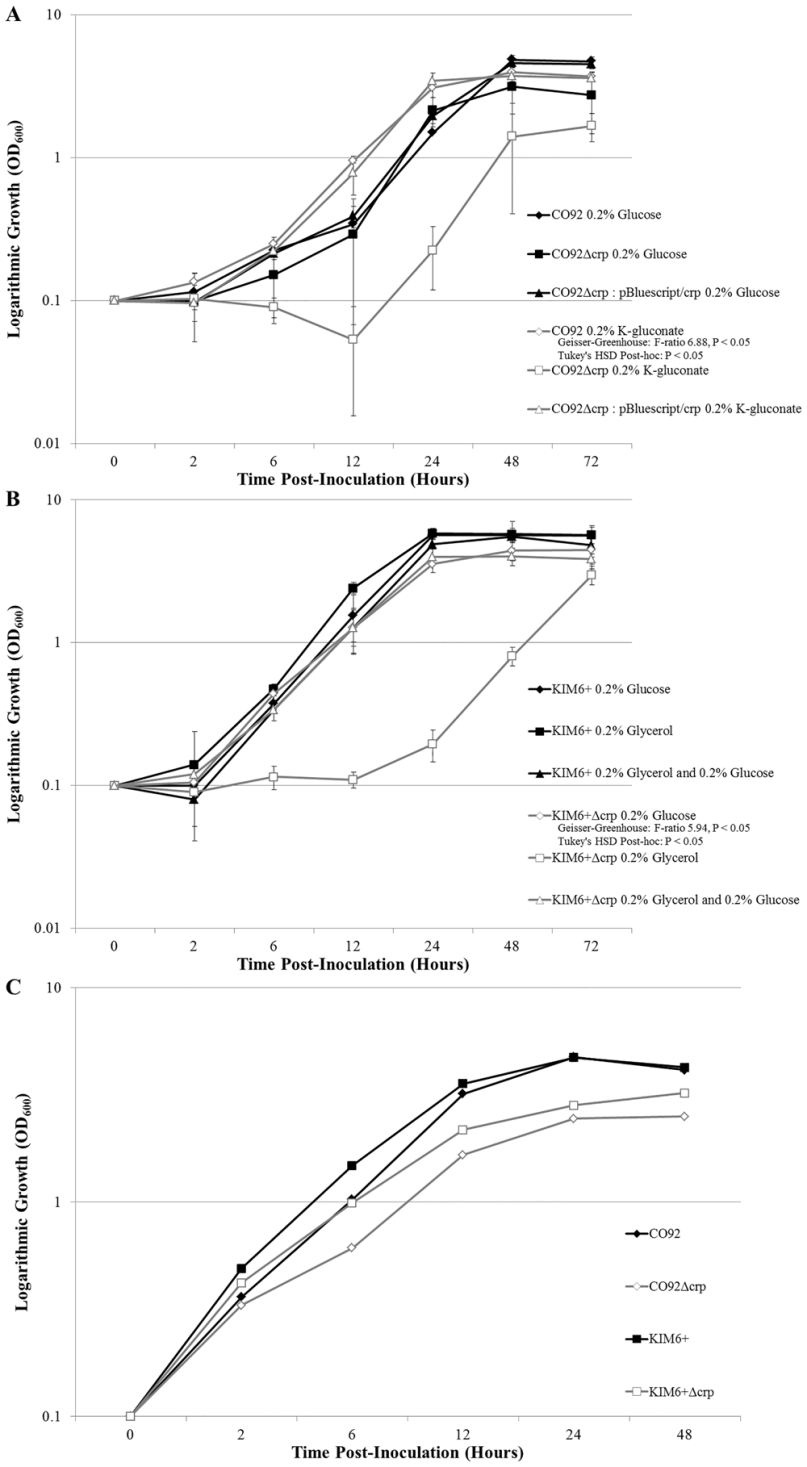
*Y*. *pestis crp*-deficient Mutant 26°C Growth Kinetics. (A) Growth curves of CO92, CO92Δ*crp*, and plasmid complemented CO92Δ*crp*: pBluescript/*crp* when grown in BCS medium supplemented with 0.2% glucose or 0.2% K-gluconate. Growth of the CO92 *crp*-deficient mutant when cultured in medium supplemented with 0.2% K-gluconate was significantly impaired relative to all other bacterial strain and media conditions (Tukey’s HSD P < 0.05). (B) Growth curves of KIM6+ and KIM6+Δ*crp* when cultured in BCS medium supplemented with either 0.2% glucose, 0.2% glycerol, or a combination of both 0.2% glucose and 0.2% glycerol. Growth of the KIM6+ *crp*-deficient mutant when cultured in medium solely supplemented with 0.2% glycerol was significantly impaired relative to all other bacterial strain and media conditions (Tukey’s HSD P < 0.05). (C) Growth kinetics of the CO92 and KIM6+ *crp*-deficient mutants and respective isogenic controls when grown in HIB medium at 26°C.

Growth kinetics of the KIM6+Δ*crp* and CO92Δ*crp* scarless deletion mutants were determined during growth at 26°C in either HIB peptide-rich medium or BCS chemically-defined medium supplemented with primary and/or alternate carbon sources (Fig 1). Growth kinetics of the KIM6+Δ*crp* and CO92Δ*crp* scarless deletion mutants were consistent with the respective isogenic controls during growth at 26°C in BCS medium supplemented with glucose. However, the Δ*crp* mutants demonstrated a stark growth defect when inoculated in BCS medium solely supplemented with alternate carbon sources, demonstrating stringent CRP-mediated carbon catabolite repression in *Y*. *pestis*. Growth of the *crp*-deficient mutants resumed approximately 24 hours post-inoculation in BCS media solely supplemented with alternate carbon sources. After 72 hours post-inoculation, the bacterial density of the KIM6+Δ*crp* and CO92Δ*crp* mutants cultured in BCS media solely supplemented with alternate carbon sources were approximately 1.9-fold and 1.8-fold less than the respective isogenic controls. Growth kinetics of the *crp*-deficient mutants demonstrated an approximate 2-fold reduction in bacterial density following 24 hours of growth in HIB medium.

**Fig 2:**
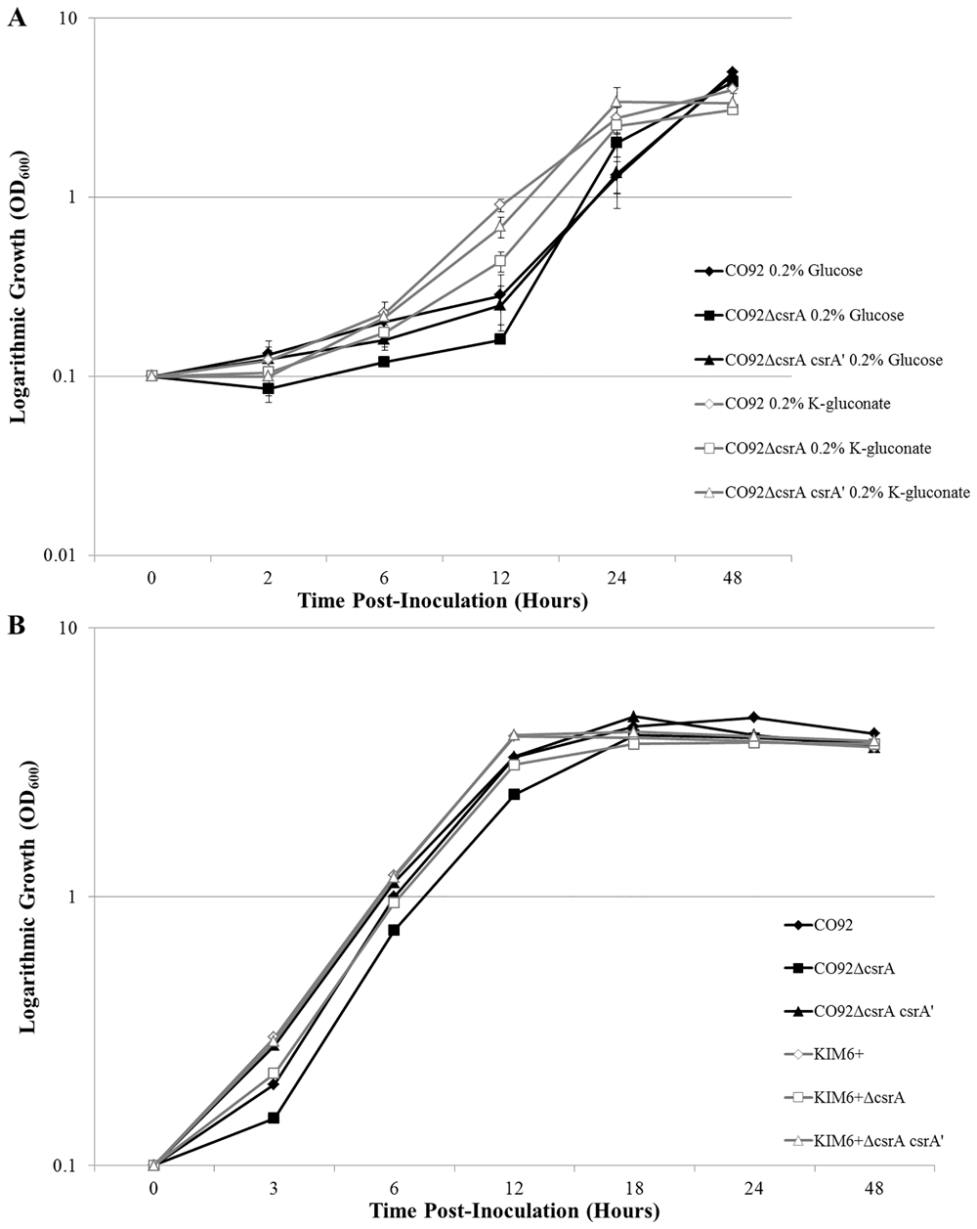
*Y*. *pestis csrA*-deficient Mutant 26°C Growth Kinetics. (A) Growth of CO92, CO92Δ*csrA*, and the chromosomal restoration mutant CO92Δ*csrA csrA*’ in BCS medium supplemented with either 0.2% glucose or 0.2% K-gluconate during incubation at 26°C. (B) Growth kinetics of the CO92 and KIM6+ *csrA*-deficient mutants and respective controls when cultured in HIB medium at 26°C. No significant alteration in growth kinetics as determined by repeated measures ANOVA was calculated among the *csrA*-deficient mutants and the respective controls, regardless of media type or available carbon source.

Deletion of *csrA* did not yield a significant alteration in 26°C growth kinetics when grown in peptide-rich HIB or chemically defined BCS medium solely supplemented with either glucose or K-gluconate (Fig 2).

### CRP Facilitates Carbon Catabolite Regulation of *Y*. *pestis* Biofilm Formation

**Fig 3:**
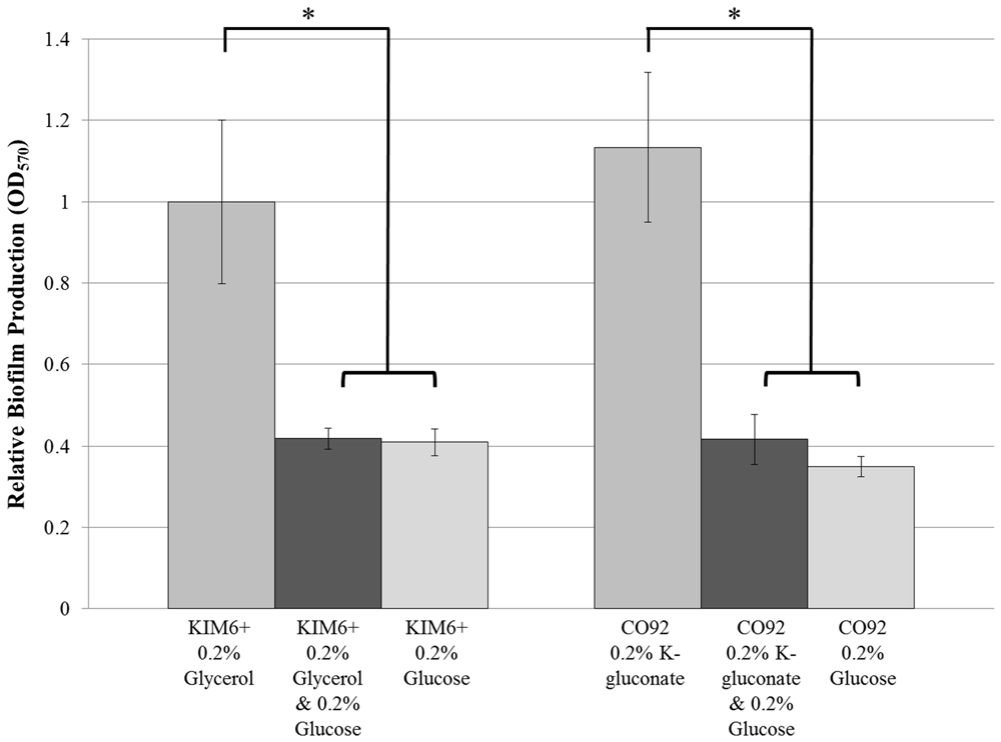
Carbon Catabolite Regulation of *Y*. *pestis* Biofilm Production. Crystal violet biofilm quantification assay of KIM6+ and CO92 when cultured in BCS medium supplemented with primary (glucose) and/or alternate (glycerol or K-gluconate) carbon sources. Error bars reflect standard deviation from the mean derived from two independent experiments, each consisting of 6 technical replicates. * P-value <0.005 determined by Tukey’s HSD post-hoc analysis.

CO92 and KIM6+ biofilm production was quantified by crystal violet assay during growth in BCS media utilizing either 0.2% glucose or 0.2% alternate carbon sources (Fig 3). After 24 hours post-inoculation, growth of *Y*. *pestis* in BCS medium solely supplemented with alternate carbon sources yielded greater than 2-fold enhanced biofilm production relative to the biofilm production in media containing glucose. Moreover, the biofilm production during growth in BCS medium containing a combination of both primary and alternate carbon sources was significantly impaired relative to the biofilm formation when grown in media solely supplemented with alternate carbon sources, indicative of a carbon catabolite repression effect. No change in growth rate was observed for either *Y*. *pestis* strain during culture in BCS medium regardless of primary or alternate carbon source supplementation. Therefore, the catabolite regulation of *Y*. *pestis* biofilm formation is independent of growth discrepancies.

**Fig 4:**
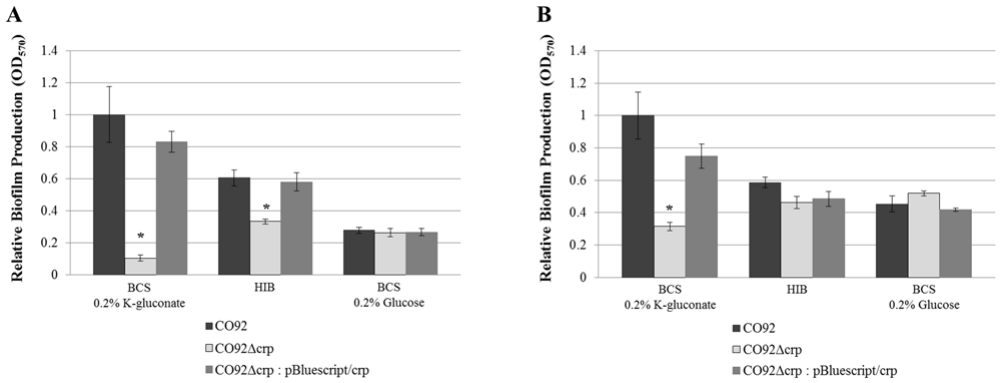
CRP Enables Robust *Y*. *pestis* Biofilm Production. Relative biofilm production of CO92, CO92Δ*crp*, and plasmid complemented CO92Δ*crp*: pBluescript/*crp* when cultured in HIB medium or BCS medium supplemented with either 0.2% K-gluconate or 0.2% glucose. (A) Following 24 hours post-inoculation. (B) After 72 hours post-inoculation. Error bars reflect standard deviation from the mean derived from two independent experiments, each consisting of 6 technical replicates. * P-value <0.005 determined by Tukey’s HSD post-hoc analysis.

The metabolism of alternate carbon sources is facilitated by CRP. Thus, the biofilm production of scarless *crp* deletion mutants constructed in both CO92 and KIM6+ backgrounds was characterized (Fig 4 and S2 Fig). When grown in the presence of glucose, deletion of *crp* had no impact upon *Y*. *pestis* biofilm production. However, biofilm formation of the *crp*-deficient mutant was significantly reduced after 24 hours post-inoculation in BCS medium solely supplemented with alternate carbon sources (Fig 4A). In order to account for the aforementioned growth defect of the *crp*-deficient mutants, biofilm production was determined at 72 hours post-inoculation biofilm production (Fig 4B). The biofilm formation of the *crp* deletion mutant was significantly impaired after 72 hours of growth in BCS medium solely supplemented with alternate carbon sources.

**Fig 5:**
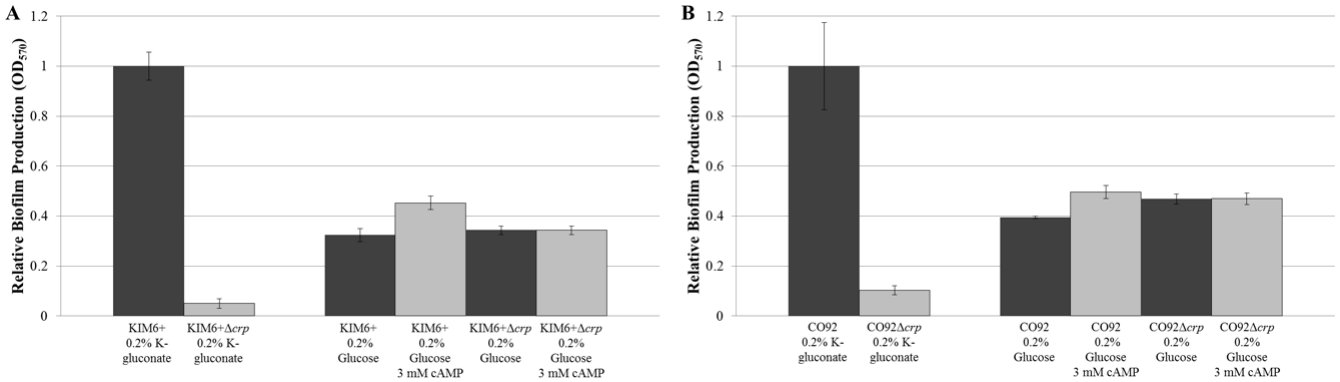
*Y*. *pestis* Biofilm Production is not Influenced by Exogenous cAMP. (A) Relative biofilm production of KIM6+ and KIM6+Δ*crp* when grown in BCS medium supplemented with 0.2% glucose containing either 3 mM cAMP prepared in K-phosphate buffer or an equal volume of plain K-phosphate buffer. (B) Relative biofilm production of CO92 and CO92Δ*crp* when grown in BCS medium supplemented with 0.2% glucose containing either 3 mM cAMP prepared in K-phosphate buffer or an equal volume of plain K-phosphate buffer. Identical source inoculum per strain was utilized for each media type. Error bars reflect standard deviation from the mean derived from two independent experiments, each consisting of 6 technical replicates. No statistically significant change in crystal violet absorption as determined by one-way ANOVA was calculated for both the *crp*-deficient mutants and the respective parental controls when cultured in media containing 3mM cAMP or an equal volume of K-phosphate buffer.

We sought to assess if cAMP-dependent CRP activation is the determinant factor defining carbon catabolite regulation of robust *Y*. *pestis* biofilm production. Supplementation of BCS media containing 0.2% glucose as the sole carbon source with 3 mM cAMP did not significantly alter biofilm formation of the *crp*-deficient mutants or the respective isogenic controls (Fig 5). Therefore, cAMP availability is not solely responsible for the potent biofilm production observed during the metabolism of alternate carbon sources. Rather, CRP-mediated metabolism of alternate carbon sources enables robust *Y*. *pestis* biofilm production.

**Fig 6:**
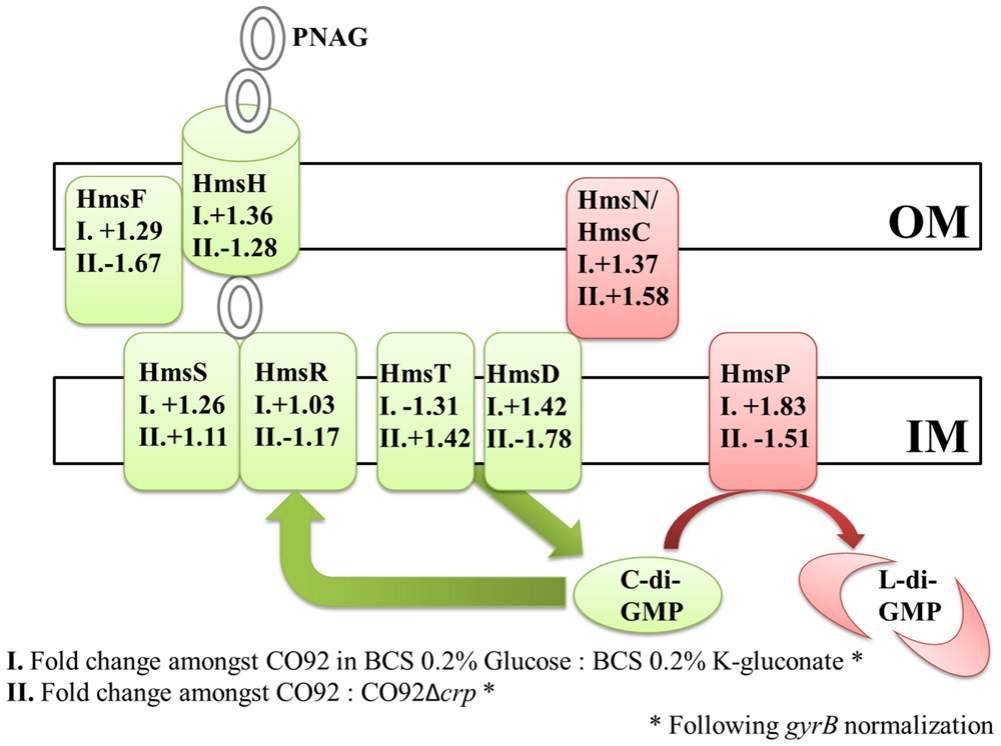
Semi-quantitative RT-PCR of the *Y*. *pestis* Hms System. Depiction of semi-quantitative RT-PCR fold changes in expression of the *Y*. *pestis* Hms biofilm formation/regulation system following normalization to the *gyrB* gene. “OM” reflects the bacterial outer membrane; whereas, “IM” indicates the inner membrane. Factors highlighted in green represent characterized pro-biofilm factors. Factors colored in red have been shown to impair biofilm production. **I**. Fold changes in mRNA abundance amongst CO92 grown in BCS medium supplemented with 0.2% glucose to CO92 cultured in BCS supplemented with 0.2% K-gluconate. **II**. Fold changes in mRNA abundance of CO92 to CO92Δ*crp* when grown in HIB medium.

CRP, a global regulator of transcription, may alter mRNA abundance of previously characterized biofilm-related genes. Transcript levels were compared among CO92 and the CO92Δ*crp* mutant during growth in HIB. Moreover, mRNA abundance was analyzed during growth of CO92 in chemically defined media utilizing either glucose or K-gluconate as the sole carbon source. Following normalization to the *gyrB* gene, semi-quantitative analyses via ImageJ software did not reveal any significant changes in relative transcript levels (Fig 6 and S3 Fig). Thus, CRP-mediated carbon catabolite modulation of biofilm formation does not alter mRNA abundance of *Y*. *pestis hms* biofilm-related genes.

### Carbon Storage Regulator Protein, CsrA, Promotes *Y*. *pestis* Biofilm Formation

**Fig 7:**
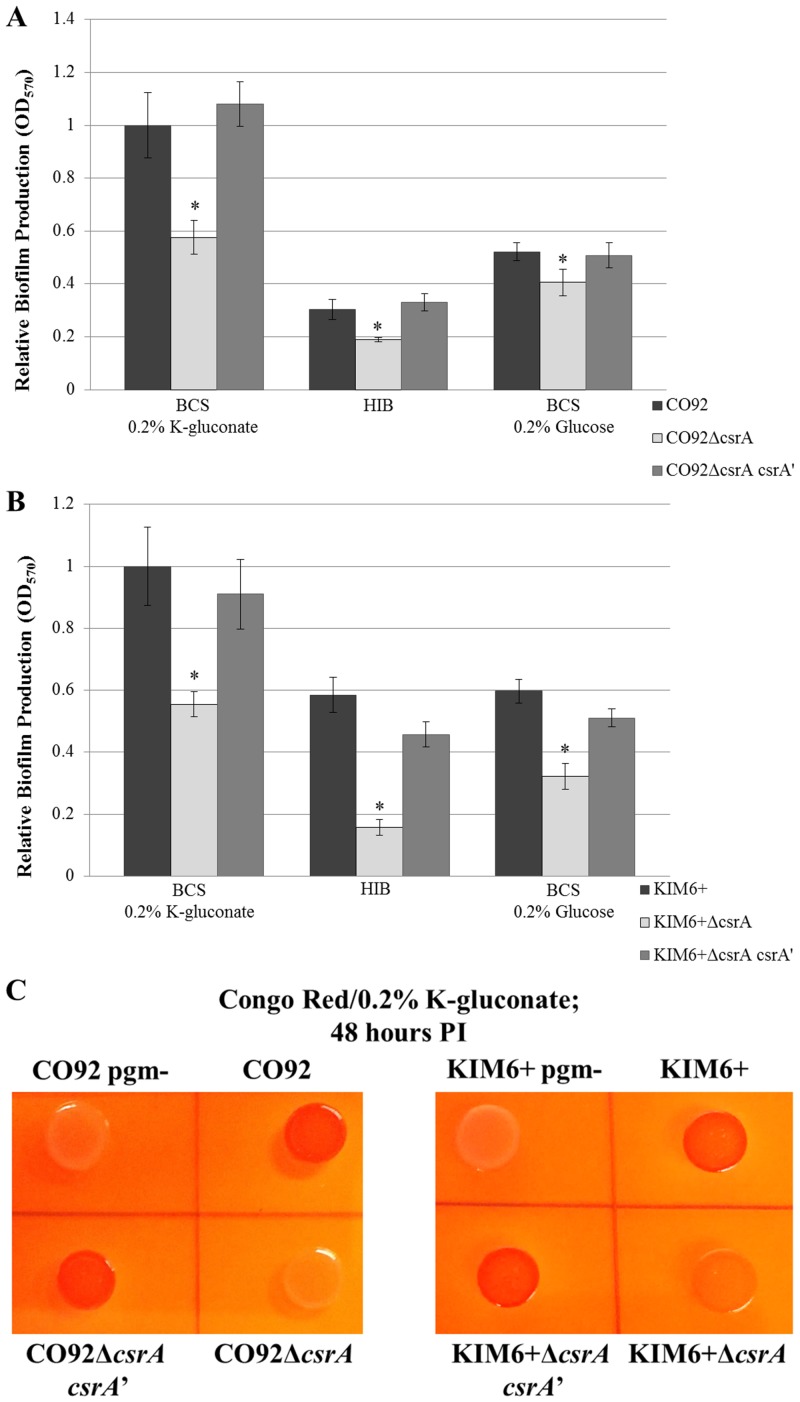
CsrA is a Positive Regulator of *Y*. *pestis* Biofilm Production. Relative biofilm production of the *csrA*-deficient mutants after 24 hours inoculation of BCS medium supplemented with 0.2% K-gluconate, HIB medium, or BCS medium supplemented with 0.2% glucose. (A) CO92, CO92Δ*csrA*, and the chromosomal restoration mutant CO92Δ*csrA csrA’*. (B) KIM6+, KIM6+Δ*csrA*, and the chromosomal restoration mutant KIM6+Δ*csrA csrA’*. Results reflect the average of two independently derived *csrA*-deficient mutants (CO92 clones 5a and 3b, and KIM6+ clones 2:14 and 4:12). Two biological replicates were assessed per mutant, each consisting of 6 technical replicates. Error bars reflect standard deviation from the mean among biological replicates. * P-value < 0.005 as determined by Tukey’s HSD post-hoc analysis. (C) Impaired Congo red assimilation of the *Y*. *pestis csrA*-deficient mutants. Phenotypic evaluation of Congo red binding of the CO92 and KIM6+ *csrA*-deficient mutants, chromosomal *csrA* restoration mutants, parental isogenic controls, and pigmentation locus negative (biofilm-deficient) mutants after 48 hours post-inoculation of Congo red plates supplemented with 0.2% K-gluconate.

To assess the impact of CsrA upon *Y*. *pestis* carbon catabolite regulation of biofilm formation, the *csrA* gene was scarlessly deleted in both the KIM6+ and CO92 *Y*. *pestis* strains. Complementation of *csrA* was afforded by scarless gene restoration in both genetic backgrounds. Biofilm formation as quantified by crystal violet assay indicated a significant reduction in biofilm formation of the *csrA*-deficient mutants relative to the isogenic control and *csrA* restoration mutant, regardless of media composition or available carbon source (Fig 7). After 24 hours post-inoculation, the relative biofilm production of the *csrA*-deficient mutant when cultured in BCS supplemented with 0.2% K-gluconate were reduced by approximately 2-fold relative to the respective control strains. Moreover, the *csrA* deletion mutants do not readily bind Congo red, indicative of severely impaired PNAG production (Fig 7C and S4 Fig). To discount potential aberrant mutations, the whole genome sequences of both the KIM6+ and CO92 *csrA*-deficient mutants were determined. Whole genome sequencing confirmed the deletion of *csrA* in both genetic backgrounds. Moreover, sequencing revealed complete retention of the pigmentation locus, including the *hmsHFRS* biofilm synthesis operon. Genomic integrity was maintained for previously characterized biofilm regulatory factors, including *hmsP*, *hmsT*, *hmsB*, *hmsC/N*, *hmsD*, *hmsE/A*, and *fur*. No conserved gaps or mutations were identified amongst the *csrA*-deficient strains (S2, S3 and S4 Tables). Considering no significant alteration in growth kinetics among the *csrA*-deficient mutants and the isogenic controls, the defect in biofilm production is independent of growth rate. Therefore, our findings demonstrate CsrA is positive regulator of *Y*. *pestis* biofilm formation.

### CsrA Enhances *Y*. *pestis* Biofilm Formation Independent of Glycogen Regulation

We sought to determine if CsrA promotes *Y*. *pestis* biofilm production through regulation of glycogen formation. Our findings demonstrate that sole deletion of *glgCAP* operon does not significantly alter *Y*. *pestis* biofilm formation (Table 2). Moreover, loss of *glgCAP* in the *csrA*-deficient background was insufficient to restore wild-type biofilm production. Similarly, deletion of *glgCAP* did not affect Congo red binding of the *csrA* deletion mutant or the parent strain (S5 Fig). These findings demonstrate that glycogen synthesis is dispensable for *Y*. *pestis* biofilm production. Therefore, the mechanism by which CsrA enhances *Y*. *pestis* biofilm production does not transpire through negative regulation of glycogen formation.

**Table 2:** Relative Crystal Violet Absorption of the CO92 Glycogen-deficient Mutants.

**BCS 0.2% K-gluconate**			
CO92	CO92Δ*csrA*	CO92Δ*glgCAP*	CO92Δ*csrA*Δ*glgCAP*
1 ± 0.074	0.65 ± 0.051*	1.02 ± 0.186	0.66 ± 0.039*
**HIB**			
CO92	CO92Δ*csrA*	CO92Δ*glgCAP*	CO92Δ*csrA*Δ*glgCAP*
0.21 ± 0.007	0.13 ± 0.005*	0.18 ± 0.007	0.13 ± 0.016*
**BCS 0.2% Glucose**			
CO92	CO92Δ*csrA*	CO92Δ*glgCAP*	CO92Δ*csrA*Δ*glgCAP*
0.61 ± 0.026	0.37 ± 0.037*	0.6 ± 0.056	0.42 ± 0.08*

* P-value < 0.05 as determined by Tukey’s HSD post-hoc analysis comparing two independent experiments, each consisting of 6 technical replicates.

### Deletion of *hmsP* Restores Biofilm Production of the *csrA*-deficient Mutant

**Fig 8:**
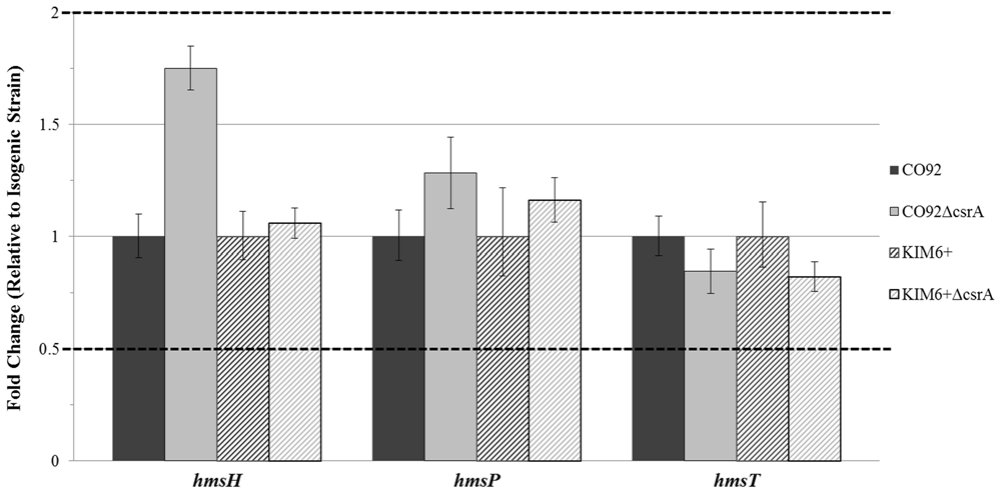
CsrA does not alter *Y*. *pestis hmsH*, *hmsP*, and *hmsT* Transcript Levels. Relative mRNA abundance of *hmsH*, *hmsP*, and *hmsT* among the *csrA*-deficient mutants and the respective parental strains calculated via the ΔΔC_T_ method in reference to the *gyrB* gene. Dashed lines reflect two-fold thresholds for alterations in relative transcript levels. No significant change in relative mRNA abundance among the deletion mutants and the respective controls were determined by one-way ANOVA comparing the average of 2 independent cDNA samples per strain, each consisting of 3 technical replicates per target.

**Fig 9:**
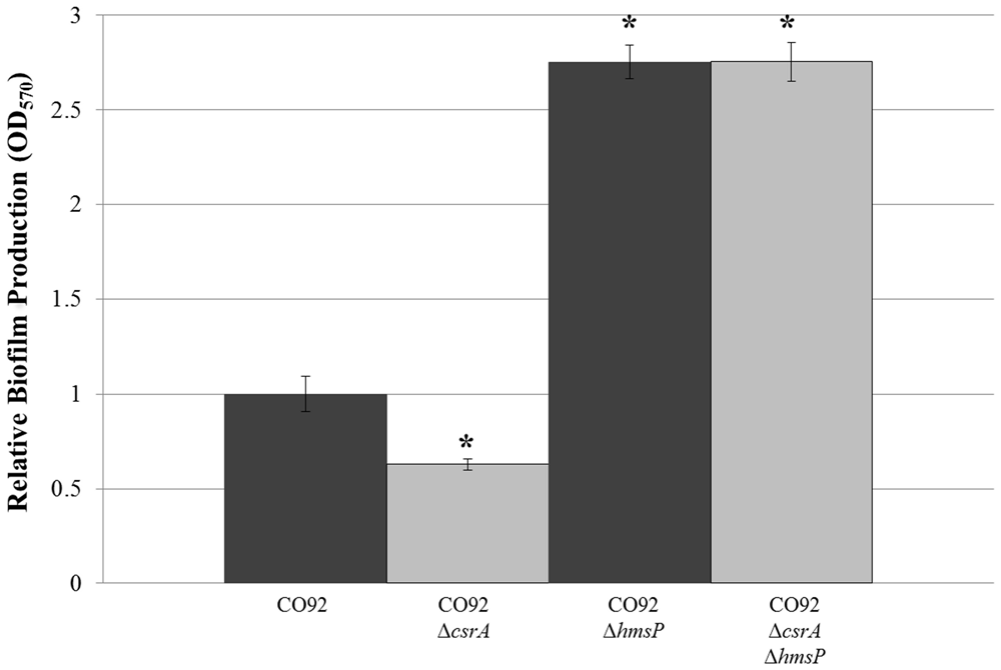
Deletion of *hmsP* Enables Excessive Biofilm Production in the *csrA*-deficient Mutant. Relative biofilm production of CO92, CO92Δ*csrA*, CO92Δ*hmsP*, and CO92Δ*csrA*Δ*hmsP* following 24 hours post-inoculation in HIB medium. Error bars reflect standard deviation from the mean of 2 independent experiments, each consisting of 6 technical replicates. * P-value < 0.005 as determined by Tukey’s HSD post-hoc analysis.

CsrA has been shown to alter the transcript stability of factors which regulate the cyclic diguanylate small molecule activator of PNAG biofilm production in *E*. *coli*; and therefore, may stimulate *Y*. *pestis* biofilm production via a similar mode of action. To assess this hypothesis, relative mRNA abundance of *hmsP*, encoding a diguanylate phosphodiesterase, and *hmsT*, encoding a diguanylate cyclase, was compared among the *csrA*-deficient mutants and parental strains. Our findings did not indicate any significant alteration in transcript levels amongst the *csrA*-deficient mutants and the respective isogenic controls (Fig 8). However, deletion of the *hmsP* gene from both wild-type CO92 and the CO92 *csrA*-deficient mutant enabled excessive biofilm production (Fig 9). Deletion of *hmsP* significantly enhanced biofilm production of the *csrA*-deficient mutant by >2.5 fold relative to the isogenic control after 24 hours of growth in HIB. Furthermore, Congo red pigmentation of the *csrA*-deficient mutant was restored upon deletion of *hmsP* (S6 Fig). Taken together, these observations suggest that CsrA may enhance *Y*. *pestis* biofilm formation through post-transcriptional regulation of cyclic diguanylate synthesis or degradation factors.

## Discussion

Within this study, our findings demonstrate that *Y*. *pestis* biofilm formation is subject to carbon catabolite regulation. The metabolism of alternate carbon sources promotes robust biofilm formation; whereas, the presence of glucose significantly impairs *Y*. *pestis* biofilm production. Considering the *Y*. *pestis* transcriptional profile during infection of the flea vector is associated with enhanced expression of alternate carbon source metabolic pathways, our findings correspond with the natural maintenance of plague in the flea midgut [16].

The metabolism of alternate carbon sources is primarily facilitated by the global transcriptional regulator, CRP. Deletion of *crp* was found to significantly impair *Y*. *pestis* growth kinetics and biofilm formation upon sole utilization of alternate carbon sources. However, media supplementation with cAMP, an activator of CRP, did not significantly alter biofilm production of the *crp*-deficient mutants or the isogenic controls. Furthermore, in accordance with findings by other investigators, CRP-mediated carbon catabolite regulation of *Y*. *pestis* biofilm formation was not associated with altered mRNA abundance of previously characterized Hms biofilm-related factors [18, 19]. Taken together, these findings suggest that CRP primarily enhances *Y*. *pestis* biofilm production by enabling the metabolism of alternate carbon sources and influencing the associated metabolic profile. CRP has been shown to stimulate ribose catabolism whilst repressing glycolysis and glycogen formation in *Y*. *pseudotuberculosis* [19]. Thus, through enhanced metabolic shunting of ribose toward PNAG or c-di-GMP biosynthesis, CRP may indirectly promote *Y*. *pestis* biofilm production (S7 Fig).

In *E*. *coli*, CsrA is known to post-transcriptionally inhibit biofilm formation via multiple modes of action. Our findings reveal that *Y*. *pestis* biofilm formation, contrary to what has been described for other *E*. *coli*, is enhanced by CsrA. *Y*. *pestis* mutants deficient in *csrA* demonstrate significantly impaired biofilm formation, irrespective of media composition or available carbon sources. Moreover, phenotypic Congo red assimilation of the *csrA* deletion mutants is drastically impaired. In fact, Congo red binding of the *csrA*-deficient mutants are comparable to the biofilm-deficient pigmentation locus negative (*hmsHFRS* negative) *Y*. *pestis*, indicative of considerable impairment of PNAG production.

A position weight matrix was derived from experimentally-characterized CsrA binding sites of *E*. *coli* [23, 28, 40, 41] (S5 Table). Utilizing the Matrix-Scan public domain software, a threshold weight score (4.0) was established through assessment of *E*. *coli pgaA* 5’ UTR which provides empirical support for predicting putative CsrA-binding sites [28]. With this strategy, putative CsrA binding sites in the *Y*. *pestis hmsH* 5’ UTR were assessed; however, only one predicted binding site was identified with an associated weight score which did not surpass the pre-determined confidence threshold (S8 Fig) [42, 43]. Therefore, dissimilar to the *E*. *coli pgaA* 5’ UTR, the *Y*. *pestis hmsH* 5’ UTR does not contain an abundance of predicted CsrA binding motifs (S8 Fig). This observation suggests CsrA may not readily associate with and impair *hmsHFRS* translation; however, does not address the mechanism by which CsrA enhances *Y*. *pestis* biofilm formation. To provide insight regarding the mechanism by which CsrA promotes *Y*. *pestis* biofilm production, we determined the impact of glycogen formation—a *csrA*-regulated process in *E*. *coli* which has been suggested to confer dual roles upon biofilm production [26, 27]. Deletion of the *glgCAP* operon from *Y*. *pestis* strain CO92 did not significantly alter crystal violet absorption and resulted in no discernable change in Congo red assimilation. Furthermore, deletion of the *glgCAP* glycogen synthesis operon from the *csrA*-deficient mutant did not restore wild-type biofilm production. Taken together, these findings demonstrate glycogen formation does not influence *Y*. *pestis* biofilm formation, indicating CsrA promotes *Y*. *pestis* biofilm formation through an alternate mode of action.

*Y*. *pestis* PNAG production is dependent upon the cyclic diguanylate small molecule activator. There are two characterized inhibitors of *Y*. *pestis* biofilm production which function through conserved mode of cyclic diguanylate regulation: 1) the Fur regulator which impairs transcription of *hmsT* encoding a diguanylate cyclase and 2) the HmsP phosphodiesterase which degrades cyclic diguanylate [14, 15, 44]. Interestingly, the 5’ UTR of *Y*. *pestis hmsP* contains multiple putative CsrA binding sites with predictive weight scores surpassing the calculated cutoff threshold, suggestive of potential CsrA interplay. (S9 Fig) [39, 44]. Comparison of *hmsP* and *hmsT* mRNA abundance among the *csrA*-deficient mutants and the respective control strains did not indicate any significant changes in transcript levels. This observation provides evidence against potential CsrA regulation of Fur. CsrA may post-transcriptionally regulate *hmsP* and/or *hmsT* expression to promote *Y*. *pestis* biofilm formation. In support of this notion, deletion of *hmsP* from a *csrA*-deficient background enabled excessive biofilm production and restored Congo red assimilation. However, further studies are necessary to confirm the role of CsrA upon *Y*. *pestis* cyclic diguanylate regulation.

Regardless of the precise mode of action, our findings establish a unique adaptation of *Y*. *pestis* CsrA regulatory cascades which enhances biofilm formation by stimulating PNAG production. It is important to note that *Y*. *pestis* is non-motile as a consequence of a frameshift mutation in the *flhD* master regulator of flagellum biosynthesis [45, 46]. Studies in *E*. *coli* and *Y*. *enterocolitica* have demonstrated that CsrA promotes motility by activating expression of the FlhDC regulators [30, 47]. Thus, through concerted ablation of motility and adaptation of PNAG synthesis regulatory cascades, the *Y*. *pestis* CsrA network has evolved to support potent biofilm production.

## Conclusions

CRP facilitates carbon catabolite regulation of *Y*. *pestis* biofilm production in which the sole metabolism of alternate carbon sources promotes robust biofilm formation; whereas, the presence of glucose impairs biofilm production. CRP does not directly control *hms* biofilm synthesis and regulation factors. Rather, we postulate CRP indirectly modulates *Y*. *pe*stis biofilm production through induction of the alternate carbon source expression profile and metabolic shunting. Unlike what has been documented for *E*. *coli*, CsrA stimulates *Y*. *pestis* biofilm production. CsrA enhances *Y*. *pestis* biofilm independent of glycogen formation which has been shown to be a dispensable factor. CsrA does not alter the mRNA abundance of *hmsP* or *hmsT* regulators of cyclic diguanylate production. However, deletion of *hmsP* in the *csrA*-deficient mutant enables excessive biofilm formation. Therefore, we conclude that CsrA may enable *Y*. *pestis* biofilm production by promoting biosynthesis or preventing degradation of the cyclic diguanylate small molecule activator of PNAG production.

## Supporting Information

S1 Fig Scarless Deletion and Chromosomal Restoration Procedure. **Step 1.** A dual selection kanamycin-resistance (KmR) and sucrose-sensitivity (SucS) cassette incorporated in pKD4 was amplified with primers containing overhangs homologous to the target gene upstream/downstream flanking sequences [33]. A gBlock DNA fragment (IDT) comprised of target gene flanking regions (scarless deletion) or the gene of interest (chromosomal restoration) was amplified in an analogous fashion. **Step 2.** Via λ-Red recombination afforded by pKD46 induction, the target gene was replaced with the KmR- SucS deletion cassette. **Step 3.** A secondary recombination event was utilized to either replace the deletion cassette with the gBlock fragment, thus excising the target gene (scarless deletion). Through additional recombination events the excised gene can be chromosomally restored through re-introduction of the selection cassette and subsequent expulsion with the gene of interest, thereby circumventing plasmid complementation.
(TIF)

S2 Fig KIM6+ *crp*-deficient Mutant Biofilm Production. Relative crystal violet absorption of KIM6+, KIM6+Δ*crp*, and plasmid complemented KIM6+Δ*crp*: pBluescript/*crp* following 24 hours post-inoculation of HIB medium or BCS medium supplemented with either 0.2% K-gluconate or 0.2% glucose incubated at 26°C. Error bars reflect standard deviation from the mean derived from two independent experiments, each consisting of 6 technical replicates. * P-value <0.005 determined by Tukey’s HSD post-hoc analysis.
(TIF)

S3 Fig Semi-Quantitative RT-PCR 2% Agarose Gels. RT-PCR of *Y*. *pestis* Hms system biofilm regulation genes visualized by EtBR.
Comparison of CO92 grown in BCS medium supplemented with either 0.2% K-gluconate or 0.2% glucose:
Row: 1 = 0.2% glucose; 2 = 0.2% K-gluconateColumns: A = *gyrB*; C = *hmsH*; E = *hmsF*; G = *hmsR*; I = *hmsS*
(Directly flanked to the right by RT negative controls)0.2% glucose: A = *gyrB*; E = *hmsP*; I = *hmsT*; M = *hmsN*; Q = *hmsD*
0.2% K-gluconate: C = *gyrB*; G = *hsmP*; K = *hmsT*; O = *hmsN*; S = *hmsD*
(Directly flanked to the right by RT negative controls)
Comparison of CO92 and CO92Δ*crp* when grown in HIB medium:
A, E, I, M, Q = CO92 *gyrB*, *hmsH*, *hmsF*, *hmsR*, *hmsS*;C, G, K, O, S = CO92Δ*crp gyrB*, *hmsH*, *hmsF*, *hmsR*, *hmsS*
(Directly flanked to the right by RT negative controls)A, E, I, M, Q = CO92 *gyrB*, *hmsP*, *hmsT*, *hmsN*, *hmsD*;C, G, K, O, S = CO92Δ*crp gyrB*, *hmsP*, *hmsT*, *hmsN*, *hmsD*
(Directly flanked to the right by RT negative controls)A, E = CO92 *gyrB*, *pla*; C, G = CO92Δ*crp gyrB*, *pla*
(Directly flanked to the right by RT negative controls).

(TIF)

S4 Fig Single Colony *csrA*-deficient Congo Red Binding Comparison. Phenotypic assessment of Congo red assimilation of KIM6+ (shown on left) and the KIM6+Δ*csrA* mutant (shown on right) single colonies after 48 hours post-inoculation of Congo red plates supplemented with 0.2% galactose incubated at 26°C.
(TIF)

S5 Fig Glycogen-deficient Mutant Congo Red Binding. Congo red binding phenotypic assessment of the Δ*glgCAP* mutants constructed in the CO92 background after 48 hours post-inoculation of Congo red plates supplemented with 0.2% K-gluconate incubated at 26°C.
(TIF)

S6 Fig *hmsP*-deficient Mutant Congo Red Binding. Congo red binding phenotypic assessment of the Δ*hmsP* mutants constructed in the CO92 background after 48 hours post-inoculation of Congo red plates supplemented with 0.2% K-gluconate incubated at 26°C.
(TIF)

S7 Fig Putative CRP Metabolic Influence upon *Yersinia* Biofilm Production. Schematic depicting the metabolic influence of CRP in *Y*. *pseudotuberculosis* [19]. Factors in green reflect CRP-induced metabolites and/or associated metabolic pathways; whereas, factors highlighted in red are impaired by CRP. CRP promotes ribose catabolism which may contribute to purine synthesis, potentially enhancing c-di-GMP production and the activation of biofilm formation. Alternately, as a concerted consequence of impaired glycogen production and glycolysis, ribose catabolism may stimulate the biosynthesis of PNAG.
(TIF)

S8 Fig *E*. *coli* and *Y*. *pestis* PNAG Biosynthesis Operon 5’ UTR Comparison. Comparison of *E*. *coli pgaA* 5’ UTR and the homologous *Y*. *pestis hmsH* 5’ UTR [28, 41]. Numerals I-VI indicate experimentally characterized CsrA interaction sites [28]. Yellow highlights reflect putative “GGA” CsrA binding motifs. Parenthetical values indicate Matrix-Scan weight scores upon assessment of the CsrA position weight matrix derived in S5 Table [42]. Assessment of experimentally-characterized CsrA-binding sites in *pgaA* 5’ UTR established a weight score confidence threshold of 4.0. Dissimilar to the *E*. *coli pgaA* 5’ UTR, the *Y*. *pestis hmsH* 5’ UTR does not encompass an abundance of putative CsrA binding motifs.
(TIF)

S9 Fig *Y*. *pestis hmsP* and *hmsT* 5’ UTR Predicted CsrA-binding sites. Analysis of *Y*. *pestis hmsP* and *hmsT* 5’ UTRs [38, 43]. Yellow highlights reflect putative “GGA” CsrA binding motifs. Parenthetical values indicate Matrix-Scan weight scores upon assessment of the CsrA position weight matrix derived in S5 Table [42]. The *hmsP* 5’ UTR contains multiple putative CsrA-binding sites which surpass the weight score confidence threshold of 4.0 obtained through assessment of the experimentally characterized *E*. *coli pgaA* 5’ UTR.
(TIF)

S1 Table List of Oligonucleotides. Detailed list of oligonucleotides utilized in this study.
(XLSX)

S2 Table Whole Genome Sequencing Coverage. Reference recovery statistics of the *Y*. *pestis csrA*-deficient mutant whole genome sequences.
(DOCX)

S3 Table Whole Genome Sequencing Mapping Gaps. Gap regions identified in the *Y*. *pestis csrA*-deficient mutant whole genome sequences.
(DOCX)

S4 Table Whole Genome Sequencing Genetic Variants. Nucleotide variants detected in the *Y*. *pestis csrA*-deficient mutant whole genome sequences.
(DOCX)

S5 Table CsrA Position Frequency Weight Matrix. Predicted CsrA binding motif derived from experimentally-characterized *E*. *coli* 5’ UTR CsrA binding sites [23, 28, 40, 41].
(DOCX)

## References

[1] Perry RD, Fetherston JD. *Yersinia pestis*—etiologic agent of plague. Clin Microbiol Rev. 1997;10(1):35–66. 8993858 10.1128/cmr.10.1.35 PMC172914

[2] World Health Organization (WHO). Human plague: review of regional morbidity and mortality, 2004–2009. Wkly Epidemiol Rec. 2009;85(6):40–5. . 20151494

[3] Eisen RJ, Bearden SW, Wilder AP, Montenieri JA, Antolin MF, Gage KL. Early-phase transmission of *Yersinia pestis* by unblocked fleas as a mechanism explaining rapidly spreading plague epizootics. Proc Natl Acad Sci U S A. 2006;103(42):15380–5. 10.1073/pnas.0606831103 17032761 PMC1592641

[4] Eisen RJ, Gage KL. Adaptive strategies of *Yersinia pestis* to persist during inter-epizootic and epizootic periods. Vet Res. 2009;40(2):1 10.1051/vetres:2008039 18803931 PMC2695026

[5] Bacot AW, Martin CJ. LXVII. Observations on the mechanism of the transmission of plague by fleas. J Hyg (Lond). 1914;13(Suppl):423–39. 20474555 PMC2167459

[6] Hinnebusch BJ. The evolution of flea-borne transmission in *Yersinia pestis* . Curr Issues Mol Biol. 2005;7(2):197–212. . 16053250

[7] Hinnebusch BJ, Erickson DL. *Yersinia pestis* biofilm in the flea vector and its role in the transmission of plague. Curr Top Microbiol Immunol. 2008;322:229–48. 18453279 10.1007/978-3-540-75418-3_11 PMC3727414

[8] Chouikha I, Hinnebusch BJ. *Yersinia*—flea interactions and the evolution of the arthropod-borne transmission route of plague. Curr Opin Microbiol. 2012;15(3):239–46. 10.1016/j.mib.2012.02.003 22406208 PMC3386424

[9] Bobrov AG, Kirillina O, Forman S, Mack D, Perry RD. Insights into *Yersinia pestis* biofilm development: topology and co-interaction of Hms inner membrane proteins involved in exopolysaccharide production. Environ Microbiol. 2008;10(6):1419–32. 10.1111/j.1462-2920.2007.01554.x . 18279344

[10] Perry RD, Pendrak ML, Schuetze P. Identification and cloning of a hemin storage locus involved in the pigmentation phenotype of *Yersinia pestis* . J Bacteriol. 1990;172(10):5929–37. 2211518 10.1128/jb.172.10.5929-5937.1990 PMC526914

[11] Bobrov AG, Kirillina O, Ryjenkov DA, Waters CM, Price PA, Fetherston JD, et al Systematic analysis of cyclic di-GMP signalling enzymes and their role in biofilm formation and virulence in *Yersinia pestis* . Mol Microbiol. 2011;79(2):533–51. 10.1111/j.1365-2958.2010.07470.x 21219468 PMC3058942

[12] Jones HA, Lillard JW, Perry RD. HmsT, a protein essential for expression of the haemin storage (Hms+) phenotype of *Yersinia pestis* . Microbiology. 1999;145 (Pt 8):2117–28. . 10463178 10.1099/13500872-145-8-2117

[13] Sun YC, Koumoutsi A, Jarrett C, Lawrence K, Gherardini FC, Darby C, et al Differential control of *Yersinia pestis* biofilm formation *in vitro* and in the flea vector by two c-di-GMP diguanylate cyclases. PLoS One. 2011;6(4):e19267 10.1371/journal.pone.0019267 21559445 PMC3084805

[14] Bobrov AG, Kirillina O, Perry RD. The phosphodiesterase activity of the HmsP EAL domain is required for negative regulation of biofilm formation in *Yersinia pestis* . FEMS Microbiol Lett. 2005;247(2):123–30. 10.1016/j.femsle.2005.04.036 15935569

[15] Kirillina O, Fetherston JD, Bobrov AG, Abney J, Perry RD. HmsP, a putative phosphodiesterase, and HmsT, a putative diguanylate cyclase, control Hms-dependent biofilm formation in *Yersinia pestis* . Mol Microbiol. 2004;54(1):75–88. 10.1111/j.1365-2958.2004.04253.x . 15458406

[16] Vadyvaloo V, Jarrett C, Sturdevant DE, Sebbane F, Hinnebusch BJ. Transit through the flea vector induces a pretransmission innate immunity resistance phenotype in *Yersinia pestis* . PLoS Pathog. 2010;6(2):e1000783 10.1371/journal.ppat.1000783 20195507 PMC2829055

[17] Busby S, Ebright RH. Transcription activation by catabolite activator protein (CAP). J Mol Biol. 1999;293(2):199–213. 10.1006/jmbi.1999.3161 . 10550204

[18] Zhan L, Han Y, Yang L, Geng J, Li Y, Gao H, et al The cyclic AMP receptor protein, CRP, is required for both virulence and expression of the minimal CRP regulon in *Yersinia pestis* biovar microtus. Infect Immun. 2008;76(11):5028–37. 10.1128/IAI.00370-08 18710863 PMC2573370

[19] Heroven AK, Sest M, Pisano F, Scheb-Wetzel M, Steinmann R, Böhme K, et al Crp induces switching of the CsrB and CsrC RNAs in *Yersinia pseudotuberculosis* and links nutritional status to virulence. Front Cell Infect Microbiol. 2012;2:158 10.3389/fcimb.2012.00158 23251905 PMC3523269

[20] Liu MY, Gui G, Wei B, Preston JF, Oakford L, Yüksel U, et al The RNA molecule CsrB binds to the global regulatory protein CsrA and antagonizes its activity in *Escherichia coli* . J Biol Chem. 1997;272(28):17502–10. . 9211896 10.1074/jbc.272.28.17502

[21] Weilbacher T, Suzuki K, Dubey AK, Wang X, Gudapaty S, Morozov I, et al A novel sRNA component of the carbon storage regulatory system of *Escherichia coli* . Mol Microbiol. 2003;48(3):657–70. . 12694612 10.1046/j.1365-2958.2003.03459.x

[22] Romeo T, Vakulskas CA, Babitzke P. Post-transcriptional regulation on a global scale: form and function of Csr/Rsm systems. Environ Microbiol. 2013;15(2):313–24. 10.1111/j.1462-2920.2012.02794.x 22672726 PMC3443267

[23] Baker CS, Morozov I, Suzuki K, Romeo T, Babitzke P. CsrA regulates glycogen biosynthesis by preventing translation of *glgC* in *Escherichia coli* . Mol Microbiol. 2002;44(6):1599–610. . 12067347 10.1046/j.1365-2958.2002.02982.x

[24] Romeo T, Gong M, Liu MY, Brun-Zinkernagel AM. Identification and molecular characterization of *csrA*, a pleiotropic gene from *Escherichia coli* that affects glycogen biosynthesis, gluconeogenesis, cell size, and surface properties. J Bacteriol. 1993;175(15):4744–55. 8393005 10.1128/jb.175.15.4744-4755.1993 PMC204926

[25] Yang H, Liu MY, Romeo T. Coordinate genetic regulation of glycogen catabolism and biosynthesis in *Escherichia coli* via the CsrA gene product. J Bacteriol. 1996;178(4):1012–7. 8576033 10.1128/jb.178.4.1012-1017.1996 PMC177760

[26] Jackson DW, Suzuki K, Oakford L, Simecka JW, Hart ME, Romeo T. Biofilm formation and dispersal under the influence of the global regulator CsrA of *Escherichia coli* . J Bacteriol. 2002;184(1):290–301. 11741870 10.1128/JB.184.1.290-301.2002 PMC134780

[27] Rahimpour M, Montero M, Almagro G, Viale AM, Sevilla Á, Cánovas M, et al GlgS, described previously as a glycogen synthesis control protein, negatively regulates motility and biofilm formation in *Escherichia coli* . Biochem J. 2013;452(3):559–73. 10.1042/BJ20130154 . 23537328

[28] Wang X, Dubey AK, Suzuki K, Baker CS, Babitzke P, Romeo T. CsrA post-transcriptionally represses *pgaABCD*, responsible for synthesis of a biofilm polysaccharide adhesin of *Escherichia coli* . Mol Microbiol. 2005;56(6):1648–63. 10.1111/j.1365-2958.2005.04648.x . 15916613

[29] Jonas K, Edwards AN, Simm R, Romeo T, Römling U, Melefors O. The RNA binding protein CsrA controls cyclic di-GMP metabolism by directly regulating the expression of GGDEF proteins. Mol Microbiol. 2008;70(1):236–57. 10.1111/j.1365-2958.2008.06411.x 18713317 PMC2735045

[30] Yakhnin AV, Baker CS, Vakulskas CA, Yakhnin H, Berezin I, Romeo T, et al CsrA activates *flhDC* expression by protecting *flhDC* mRNA from RNase E-mediated cleavage. Mol Microbiol. 2013;87(4):851–66. 10.1111/mmi.12136 23305111 PMC3567230

[31] Datsenko KA, Wanner BL. One-step inactivation of chromosomal genes in *Escherichia coli* K-12 using PCR products. Proc Natl Acad Sci U S A. 2000;97(12):6640–5. 10.1073/pnas.120163297 10829079 PMC18686

[32] Sun W, Wang S, Curtiss R. Highly efficient method for introducing successive multiple scarless gene deletions and markerless gene insertions into the *Yersinia pestis* chromosome. Appl Environ Microbiol. 2008;74(13):4241–5. 10.1128/AEM.00940-08 18487404 PMC2446500

[33] Willias SP, Chauhan S, Motin VL. Functional characterization of *Yersinia pestis* aerobic glycerol metabolism. Microb Pathog. 2014;76:33–43. 10.1016/j.micpath.2014.08.010 25220241 PMC4250381

[34] Fowler JM, Brubaker RR. Physiological basis of the low calcium response in *Yersinia pestis* . Infect Immun. 1994;62(12):5234–41. 7960099 10.1128/iai.62.12.5234-5241.1994 PMC303259

[35] Perry RD, Bobrov AG, Kirillina O, Jones HA, Pedersen L, Abney J, et al Temperature regulation of the hemin storage (Hms+) phenotype of *Yersinia pestis* is posttranscriptional. J Bacteriol. 2004;186(6):1638–47. 14996794 10.1128/JB.186.6.1638-1647.2004 PMC355957

[36] Surgalla MJ, Beesley ED. Congo red-agar plating medium for detecting pigmentation in *Pasteurella pestis* . Appl Microbiol. 1969;18(5):834–7. 5370459 10.1128/am.18.5.834-837.1969 PMC378096

[37] Cathelyn JS, Crosby SD, Lathem WW, Goldman WE, Miller VL. RovA, a global regulator of *Yersinia pestis*, specifically required for bubonic plague. Proc Natl Acad Sci U S A. 2006;103(36):13514–9. 10.1073/pnas.0603456103 16938880 PMC1569194

[38] Girish V, Vijayalakshmi A. Affordable image analysis using NIH Image/ImageJ. Indian J Cancer. 2004;41(1):47 . 15105580

[39] Bellows LE, Koestler BJ, Karaba SM, Waters CM, Lathem WW. Hfq-dependent, co-ordinate control of cyclic diguanylate synthesis and catabolism in the plague pathogen *Yersinia pestis* . Mol Microbiol. 2012;86(3):661–74. 10.1111/mmi.12011 22924957 PMC3480973

[40] Baker CS, Eöry LA, Yakhnin H, Mercante J, Romeo T, Babitzke P. CsrA inhibits translation initiation of *Escherichia coli hfq* by binding to a single site overlapping the Shine-Dalgarno sequence. J Bacteriol. 2007;189(15):5472–81. 10.1128/JB.00529-07 17526692 PMC1951803

[41] Dubey AK, Baker CS, Suzuki K, Jones AD, Pandit P, Romeo T, et al CsrA regulates translation of the *Escherichia coli* carbon starvation gene, *cstA*, by blocking ribosome access to the *cstA* transcript. J Bacteriol. 2003;185(15):4450–60. 12867454 10.1128/JB.185.15.4450-4460.2003 PMC165747

[42] Fang N, Yang H, Fang H, Liu L, Zhang Y, Wang L, et al RcsAB is a major repressor of *Yersinia* biofilm development through directly acting on *hmsCDE*, *hmsT*, and *hmsHFRS* . Sci Rep. 2015;5:9566 10.1038/srep09566 25828910 PMC4381331

[43] Turatsinze JV, Thomas-Chollier M, Defrance M, van Helden J. Using RSAT to scan genome sequences for transcription factor binding sites and *cis*-regulatory modules. Nat Protoc. 2008;3(10):1578–88. 10.1038/nprot.2008.97 . 18802439

[44] Sun F, Gao H, Zhang Y, Wang L, Fang N, Tan Y, et al Fur is a repressor of biofilm formation in *Yersinia pestis* . PLoS One. 2012;7(12):e52392 10.1371/journal.pone.0052392 23285021 PMC3528687

[45] Young GM, Smith MJ, Minnich SA, Miller VL. The *Yersinia enterocolitica* motility master regulatory operon, *flhDC*, is required for flagellin production, swimming motility, and swarming motility. J Bacteriol. 1999;181(9):2823–33. 10217774 10.1128/jb.181.9.2823-2833.1999 PMC93725

[46] Chain PS, Carniel E, Larimer FW, Lamerdin J, Stoutland PO, Regala WM, et al Insights into the evolution of *Yersinia pestis* through whole-genome comparison with *Yersinia pseudotuberculosis* . Proc Natl Acad Sci U S A. 2004;101(38):13826–31. 10.1073/pnas.0404012101 15358858 PMC518763

[47] LeGrand K, Petersen S, Zheng Y, Liu KK, Ozturk G, Chen JY, et al CsrA impacts survival of *Yersinia enterocolitica* by affecting a myriad of physiological activities. BMC Microbiol. 2015;15(1):31 10.1186/s12866-015-0343-6 25885058 PMC4336687

[48] Doll JM, Zeitz PS, Ettestad P, Bucholtz AL, Davis T, Gage K. Cat-transmitted fatal pneumonic plague in a person who traveled from Colorado to Arizona. Am J Trop Med Hyg. 1994;51(1):109–14. . 8059908 10.4269/ajtmh.1994.51.109

