# Supplementary figures and images for: CRP-Mediated Carbon Catabolite Regulation of Yersinia pestis Biofilm Formation Is Enhanced by the Carbon Storage Regulator Protein, CsrA
Source: PLoS One. 2015 Aug 25;10(8):e0135481. doi: 10.1371/journal.pone.0135481 (PMC4549057; doi:10.1371/journal.pone.0135481)

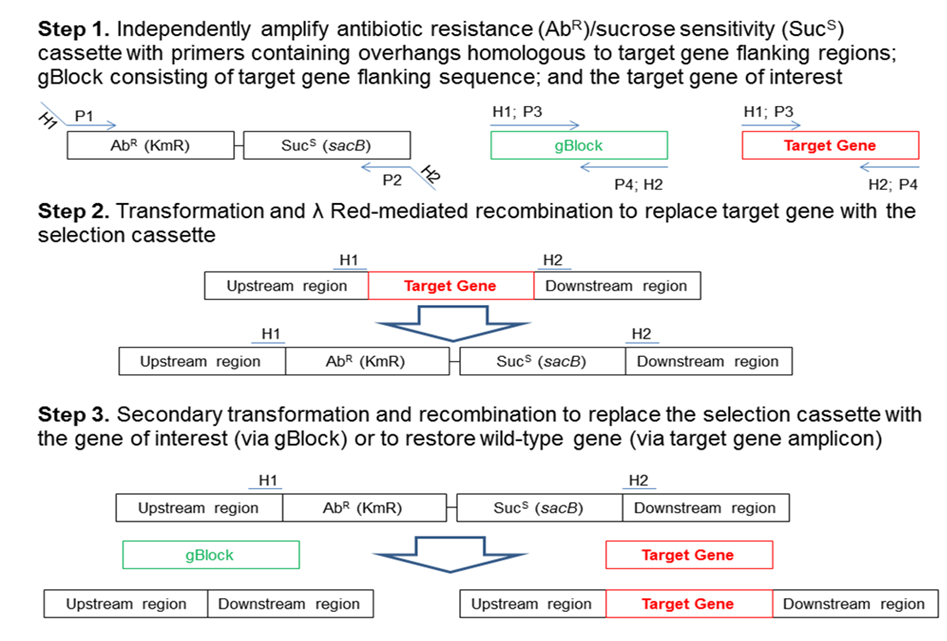

Supplement: S1 Fig — Step 1. A dual selection kanamycin-resistance (KmR) and sucrose-sensitivity (SucS) cassette incorporated in pKD4 was amplified with primers containing overhangs homologous to the target gene upstream/downstream flanking sequences [33]. A gBlock DNA fragment (IDT) comprised of target gene flanking regions (scarless deletion) or the gene of interest (chromosomal restoration) was amplified in an analogous fashion. Step 2. Via λ-Red recombination afforded by pKD46 induction, the target gene was replaced with the KmR- SucS deletion cassette. Step 3. A secondary recombination event was utilized to either replace the deletion cassette with the gBlock fragment, thus excising the target gene (scarless deletion). Through additional recombination events the excised gene can be chromosomally restored through re-introduction of the selection cassette and subsequent expulsion with the gene of interest, thereby circumventing plasmid complementation. (TIF) [file pone.0135481.s001.tif]

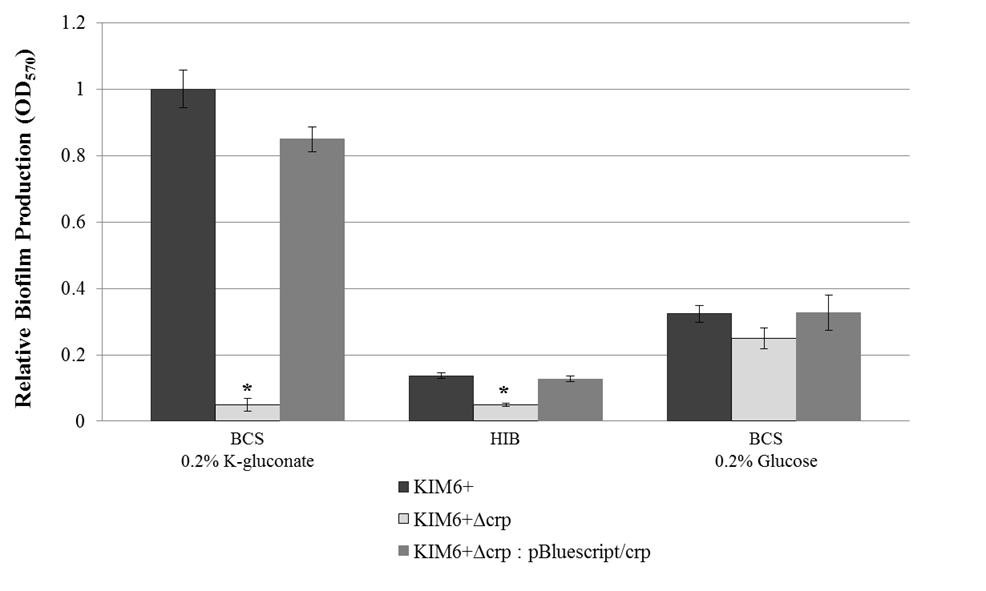

Supplement: S2 Fig — Relative crystal violet absorption of KIM6+, KIM6+Δcrp, and plasmid complemented KIM6+Δcrp: pBluescript/crp following 24 hours post-inoculation of HIB medium or BCS medium supplemented with either 0.2% K-gluconate or 0.2% glucose incubated at 26°C. Error bars reflect standard deviation from the mean derived from two independent experiments, each consisting of 6 technical replicates. * P-value <0.005 determined by Tukey’s HSD post-hoc analysis. (TIF) [file pone.0135481.s002.tif]

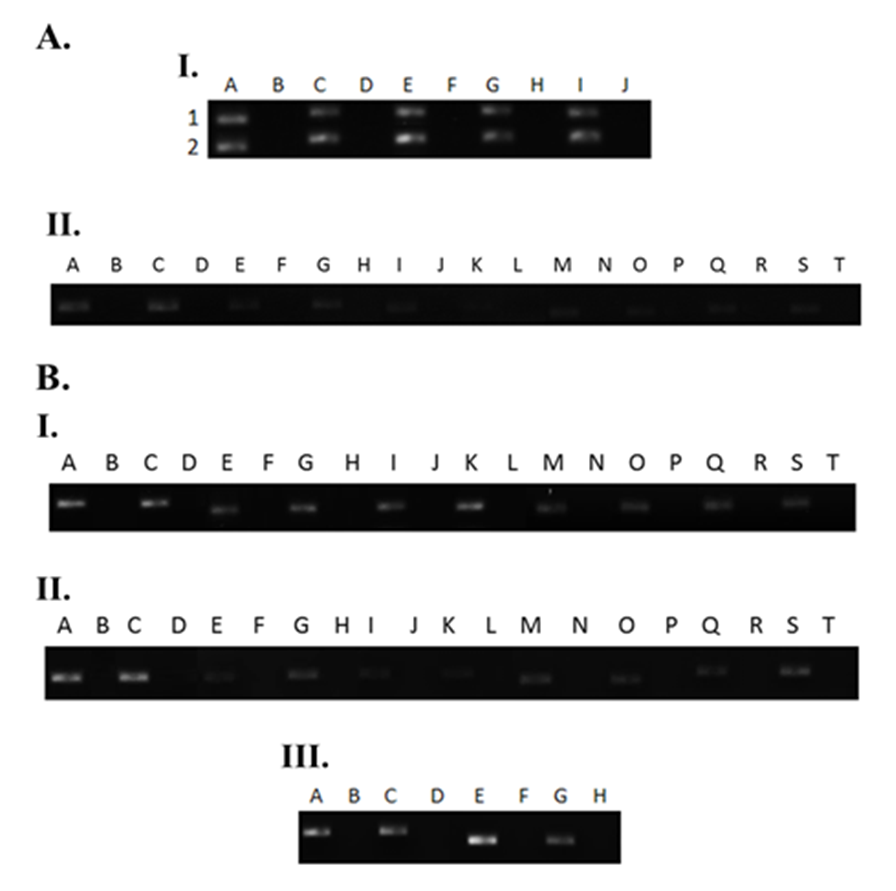

Supplement: S3 Fig — RT-PCR of Y. pestis Hms system biofilm regulation genes visualized by EtBR. Comparison of CO92 grown in BCS medium supplemented with either 0.2% K-gluconate or 0.2% glucose: Row: 1 = 0.2% glucose; 2 = 0.2% K-gluconateColumns: A = gyrB; C = hmsH; E = hmsF; G = hmsR; I = hmsS (Directly flanked to the right by RT negative controls)0.2% glucose: A = gyrB; E = hmsP; I = hmsT; M = hmsN; Q = hmsD 0.2% K-gluconate: C = gyrB; G = hsmP; K = hmsT; O = hmsN; S = hmsD (Directly flanked to the right by RT negative controls) Comparison of CO92 and CO92Δcrp when grown in HIB medium: A, E, I, M, Q = CO92 gyrB, hmsH, hmsF, hmsR, hmsS;C, G, K, O, S = CO92Δcrp gyrB, hmsH, hmsF, hmsR, hmsS (Directly flanked to the right by RT negative controls)A, E, I, M, Q = CO92 gyrB, hmsP, hmsT, hmsN, hmsD;C, G, K, O, S = CO92Δcrp gyrB, hmsP, hmsT, hmsN, hmsD (Directly flanked to the right by RT negative controls)A, E = CO92 gyrB, pla; C, G = CO92Δcrp gyrB, pla (Directly flanked to the right by RT negative controls). (TIF) [file pone.0135481.s003.tif]

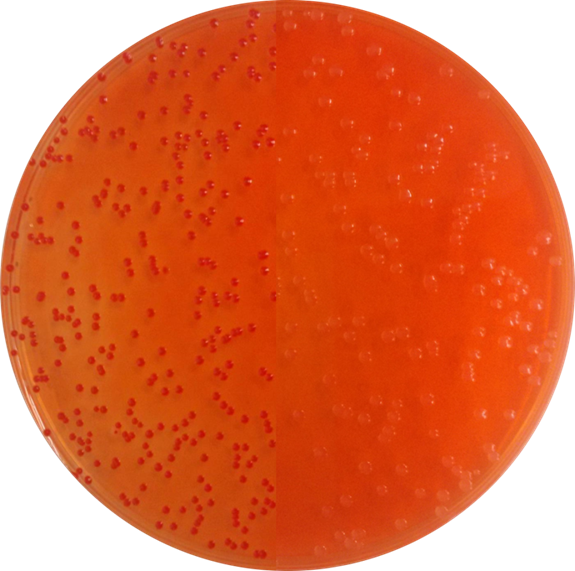

Supplement: S4 Fig — Phenotypic assessment of Congo red assimilation of KIM6+ (shown on left) and the KIM6+ΔcsrA mutant (shown on right) single colonies after 48 hours post-inoculation of Congo red plates supplemented with 0.2% galactose incubated at 26°C. (TIF) [file pone.0135481.s004.tif]

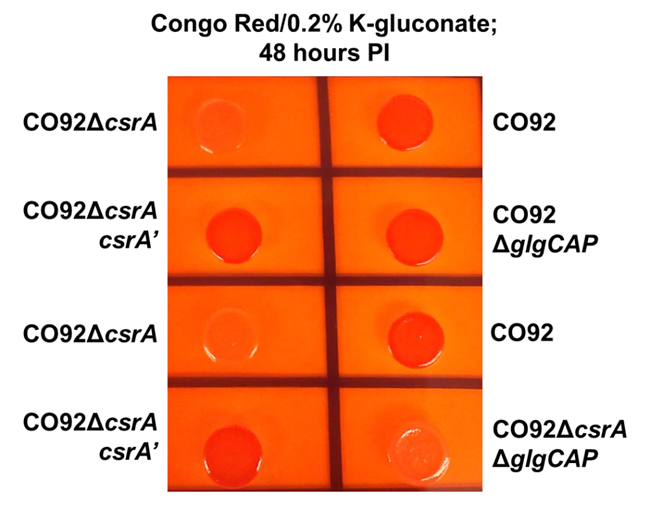

Supplement: S5 Fig — Congo red binding phenotypic assessment of the ΔglgCAP mutants constructed in the CO92 background after 48 hours post-inoculation of Congo red plates supplemented with 0.2% K-gluconate incubated at 26°C. (TIF) [file pone.0135481.s005.tif]

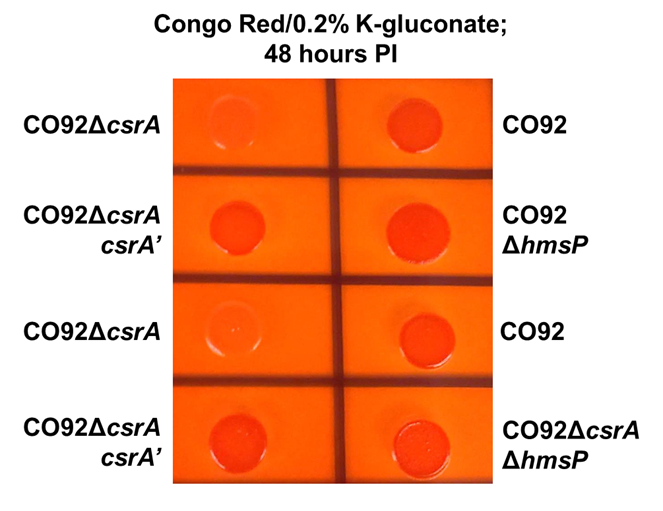

Supplement: S6 Fig — Congo red binding phenotypic assessment of the ΔhmsP mutants constructed in the CO92 background after 48 hours post-inoculation of Congo red plates supplemented with 0.2% K-gluconate incubated at 26°C. (TIF) [file pone.0135481.s006.tif]

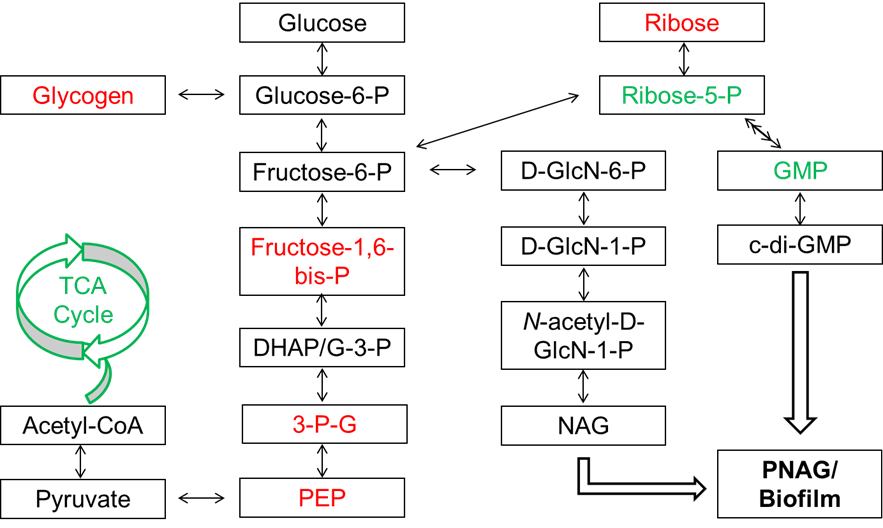

Supplement: S7 Fig — Schematic depicting the metabolic influence of CRP in Y. pseudotuberculosis [19]. Factors in green reflect CRP-induced metabolites and/or associated metabolic pathways; whereas, factors highlighted in red are impaired by CRP. CRP promotes ribose catabolism which may contribute to purine synthesis, potentially enhancing c-di-GMP production and the activation of biofilm formation. Alternately, as a concerted consequence of impaired glycogen production and glycolysis, ribose catabolism may stimulate the biosynthesis of PNAG. (TIF) [file pone.0135481.s007.tif]

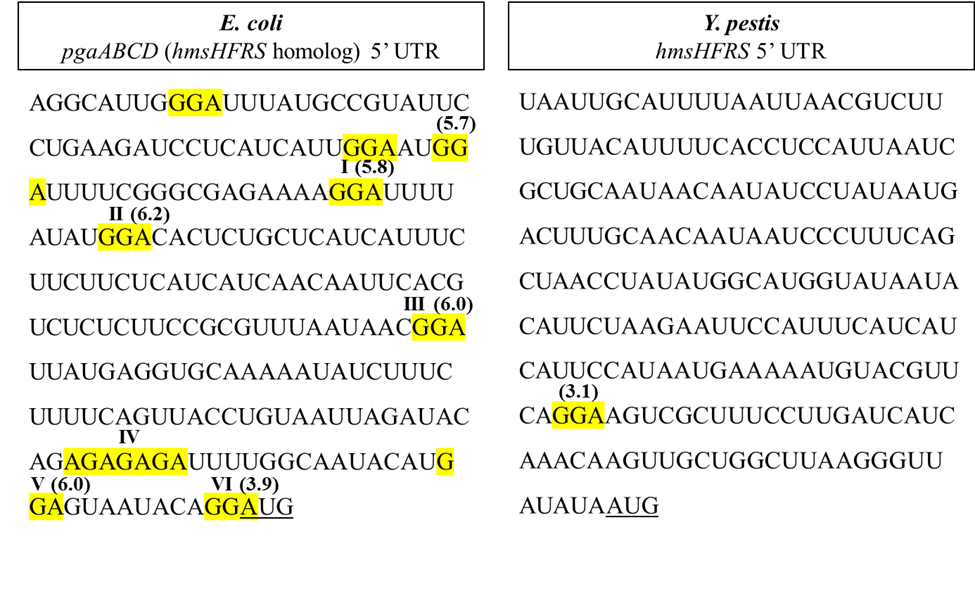

Supplement: S8 Fig — Comparison of E. coli pgaA 5’ UTR and the homologous Y. pestis hmsH 5’ UTR [28, 41]. Numerals I-VI indicate experimentally characterized CsrA interaction sites [28]. Yellow highlights reflect putative “GGA” CsrA binding motifs. Parenthetical values indicate Matrix-Scan weight scores upon assessment of the CsrA position weight matrix derived in S5 Table [42]. Assessment of experimentally-characterized CsrA-binding sites in pgaA 5’ UTR established a weight score confidence threshold of 4.0. Dissimilar to the E. coli pgaA 5’ UTR, the Y. pestis hmsH 5’ UTR does not encompass an abundance of putative CsrA binding motifs. (TIF) [file pone.0135481.s008.tif]

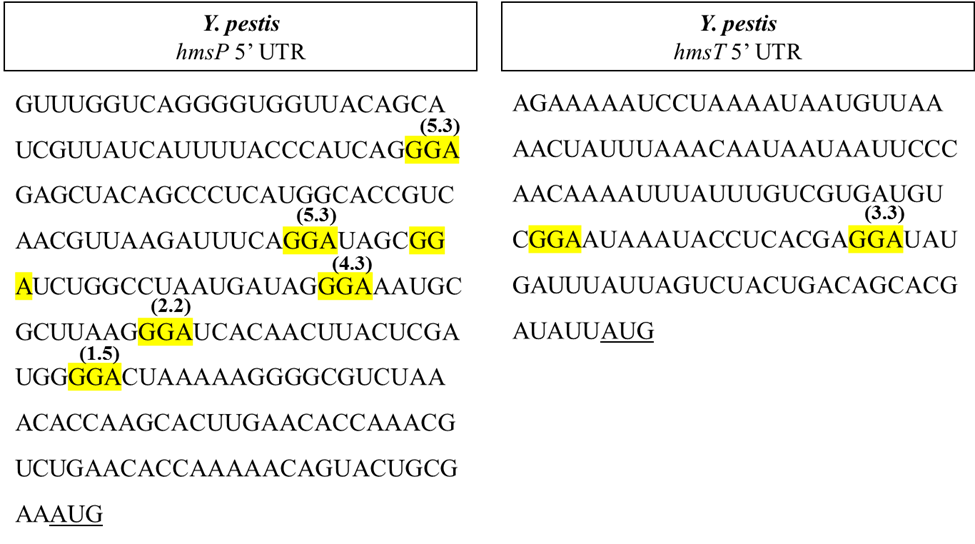

Supplement: S9 Fig — Analysis of Y. pestis hmsP and hmsT 5’ UTRs [38, 43]. Yellow highlights reflect putative “GGA” CsrA binding motifs. Parenthetical values indicate Matrix-Scan weight scores upon assessment of the CsrA position weight matrix derived in S5 Table [42]. The hmsP 5’ UTR contains multiple putative CsrA-binding sites which surpass the weight score confidence threshold of 4.0 obtained through assessment of the experimentally characterized E. coli pgaA 5’ UTR. (TIF) [file pone.0135481.s009.tif]
