# Supplementary material for: CRP-Mediated Carbon Catabolite Regulation of Yersinia pestis Biofilm Formation Is Enhanced by the Carbon Storage Regulator Protein, CsrA
Source: PLoS One. 2015 Aug 25;10(8):e0135481. doi: 10.1371/journal.pone.0135481 (PMC4549057; doi:10.1371/journal.pone.0135481)
Supplement: S2 Table — Reference recovery statistics of the Y. pestis csrA-deficient mutant whole genome sequences. (DOCX) [file pone.0135481.s011.docx]

**S2 Table.** Whole Genome Sequencing Coverage.

|  | **Mapped**  **reads** | **Reference**  **Recovery**  **(%)** | **Average Fold Coverage**  **(X)** | **Fold**  **Standard**  **Deviation** |
| --- | --- | --- | --- | --- |
| **Ref: NC_003143** |  |  |  |  |
| CO92  ∆*csrA* 5a | 4831565 (67.5%) | 99.99 | 244.63 | 109.35 |
| **Ref: NC_004088** |  |  |  |  |
| KIM6+  ∆*csrA* 2:14 | 6084441 (74.9%) | 99.99 | 312 | 83.14 |
