# Supplementary material for: CRP-Mediated Carbon Catabolite Regulation of Yersinia pestis Biofilm Formation Is Enhanced by the Carbon Storage Regulator Protein, CsrA
Source: PLoS One. 2015 Aug 25;10(8):e0135481. doi: 10.1371/journal.pone.0135481 (PMC4549057; doi:10.1371/journal.pone.0135481)
Supplement: S3 Table — Gap regions identified in the Y. pestis csrA-deficient mutant whole genome sequences. (DOCX) [file pone.0135481.s012.docx]

**S3 Table.** Whole Genome Sequencing Mapping Gaps.

| **Sample** | **Gap**  **Start*** | **Gap**  **End*** | **Gap**  **Size** | **CDS**  **Start*** | **CDS**  **End*** | **CDS**  **Product** |
| --- | --- | --- | --- | --- | --- | --- |
| **Ref: NC_003143** |  |  |  |  |  |  |
| CO92  ∆*csrA* 5a | 2293680 | 2293697 | 18 |  |  |  |
| CO92∆*csrA* 5a | 3683281 | 3683464 | 184 | 3683279 | 3683464 | YPO3304:  carbon storage regulator |
| **Ref: NC_004088** |  |  |  |  |  |  |
| KIM6∆+  *csrA* 2:14 | 54345 | 54359 | 15 | 53277 | 54503 | y0040:  hypothetical protein |
| KIM6+  ∆*csrA* 2:14 | 995115 | 995298 | 184 | 995115 | 995300 | y0884:  carbon storage regulator |
| KIM6+  ∆*csrA* 2:14 | 2870463 | 2870485 | 23 | 2867409 | 2870561 | y2605:adhesin |

* Coordinate of the reference genome.
