# Supplementary material for: CRP-Mediated Carbon Catabolite Regulation of Yersinia pestis Biofilm Formation Is Enhanced by the Carbon Storage Regulator Protein, CsrA
Source: PLoS One. 2015 Aug 25;10(8):e0135481. doi: 10.1371/journal.pone.0135481 (PMC4549057; doi:10.1371/journal.pone.0135481)
Supplement: S4 Table — Nucleotide variants detected in the Y. pestis csrA-deficient mutant whole genome sequences. (DOCX) [file pone.0135481.s013.docx]

**S4 Table.** Whole Genome Sequencing Genetic Variants.

| **Sample** | **Ref.**  **Position** * | **Ref.**  **Seq.** | **Var.**  **Seq.** | **Type** | **CDS**  **Start** * | **CDS**  **End** * | **Product** |
| --- | --- | --- | --- | --- | --- | --- | --- |
| **Ref: NC_003143** |  |  |  |  |  |  |  |
| CO92  ∆*csrA* 5a | 1234971 | G | GA | Ins. | 1234752 | 1235063 | YPO1087:  putative prophage protein |
| **Ref: NC_004088** |  |  |  |  |  |  |  |
| KIM6+  ∆*csrA* 2:14 | 2006621 | TAA | TA | Del. |  |  | Intergenic region |
| KIM6+  ∆*csrA* 2:14 | 2959406 | CGGG | CGG | Del. | 2959360 | 2959551 | y2681:  hypothetical protein |
| KIM6+  ∆*csrA* 2:14 | 2006616 | T | C | SNP |  |  | Intergenic region |
| KIM6+  ∆*csrA* 2:14 | 3787395 | T | C | SNP |  |  | Intergenic region |

* Coordinate of the reference genome.

Ref.: reference genome; Var.: Variant; Seq.: Sequence; Ins.: Insertion; Del.: Deletion
