# Supplementary material for: CRP-Mediated Carbon Catabolite Regulation of Yersinia pestis Biofilm Formation Is Enhanced by the Carbon Storage Regulator Protein, CsrA
Source: PLoS One. 2015 Aug 25;10(8):e0135481. doi: 10.1371/journal.pone.0135481 (PMC4549057; doi:10.1371/journal.pone.0135481)
Supplement: S5 Table — Predicted CsrA binding motif derived from experimentally-characterized E. coli 5’ UTR CsrA binding sites [23, 28, 40, 41]. (DOCX) [file pone.0135481.s014.docx]

**S5 Table.** CsrA Position Frequency Weight Matrix. CsrA binding motif derived from experimentally-characterized CsrA binding sites in 5’ UTRs in *E. coli* [23, 28, 40, 41].

**G G A**

**a |** 4 10 4 0 0 12 2 6

**c |** 4 2 3 0 0 0 3 0

**g |** 0 0 1 12 12 0 2 1

**t |** 4 0 4 0 0 0 5 5
